# Catalysis tuned for metabolic sensing: dynamics and mechanism of the RNA methyltransferase METTL16

**DOI:** 10.1039/d6cb00242k

**Published:** 2026-10-07

**Authors:** Fiona Stamm, Zhongyi Wan, Pablo Andrés Vargas-Rosales, Ivan Corbeski, Emmanuelle Braud, Laura Iannazzo, Qiang Cui, Amedeo Caflisch

**Affiliations:** a Department of Biochemistry, University of Zurich Winterthurerstrasse 190 8057 Zürich Switzerland caflisch@bioc.uzh.ch; b Department of Chemistry, Boston University 590 Commonwealth Ave. Boston MA 02215 USA qiangcui@bu.edu; c Laboratoire de Chimie et Biochimie Pharmacologiques et Toxicologiques, Université Paris Cité, CNRS Paris France

## Abstract

The methyltransferase-like protein 16 (METTL16) is the writer enzyme that methylates adenosine to form N6-methyladenosine (m6A) on U6 spliceosomal RNA and MAT2A mRNA. The latter encodes the enzyme responsible for the synthesis of *S*-adenosyl methionine (SAM). Since methylation influences the stability of MAT2A mRNA, METTL16 is the sensor of SAM in the cell. Here, we investigate the (co)substrate recognition and catalytic mechanism of METTL16 by a combination of *in vitro* and simulation methods. We first solved the crystal structures of METTL16 in complex with bisubstrate analogs (BAs), conjugates of a SAM-like moiety connected to the N6-atom of adenosine. The METTL16/BA crystal structures provide an atomistic picture of a transition-state mimic of methyl transfer. Molecular dynamics (MD) simulations started from complexes with oligonucleotide-RNA modeled on the METTL16/BAs structures show rapid unbinding of SAM. The presence of the MAT2A hairpin 1 RNA stabilizes SAM in its recognition pocket, and substantially reduces the intrinsic flexibility of the R-loop and N-terminal segment of METTL16. Quantum mechanics/molecular mechanics (QM/MM) free energy calculations provide evidence that methyl transfer occurs without prior deprotonation of adenosine-N6. Furthermore, the simulations indicate that electrostatic interactions between the charged groups of SAM and the catalytic pocket are less pronounced for METTL16 than for METTL3–14, which is the main m6A-RNA writer enzyme. The multidisciplinary approach used here sheds light on the intrinsic plasticity of METTL16, the rigidification upon RNA-substrate binding, the catalytic mechanism, and (co)product release. Overall, our study suggests that the latter step is rate-limiting.

## Abstract 2

The structures of methyltransferase-like protein 16 (METTL16) in complex with bisubstrate analogs which mimic the transition state are integrated with molecular dynamics and quantum mechanical simulations to describe its catalytic mechanism.

## Graphical abstract

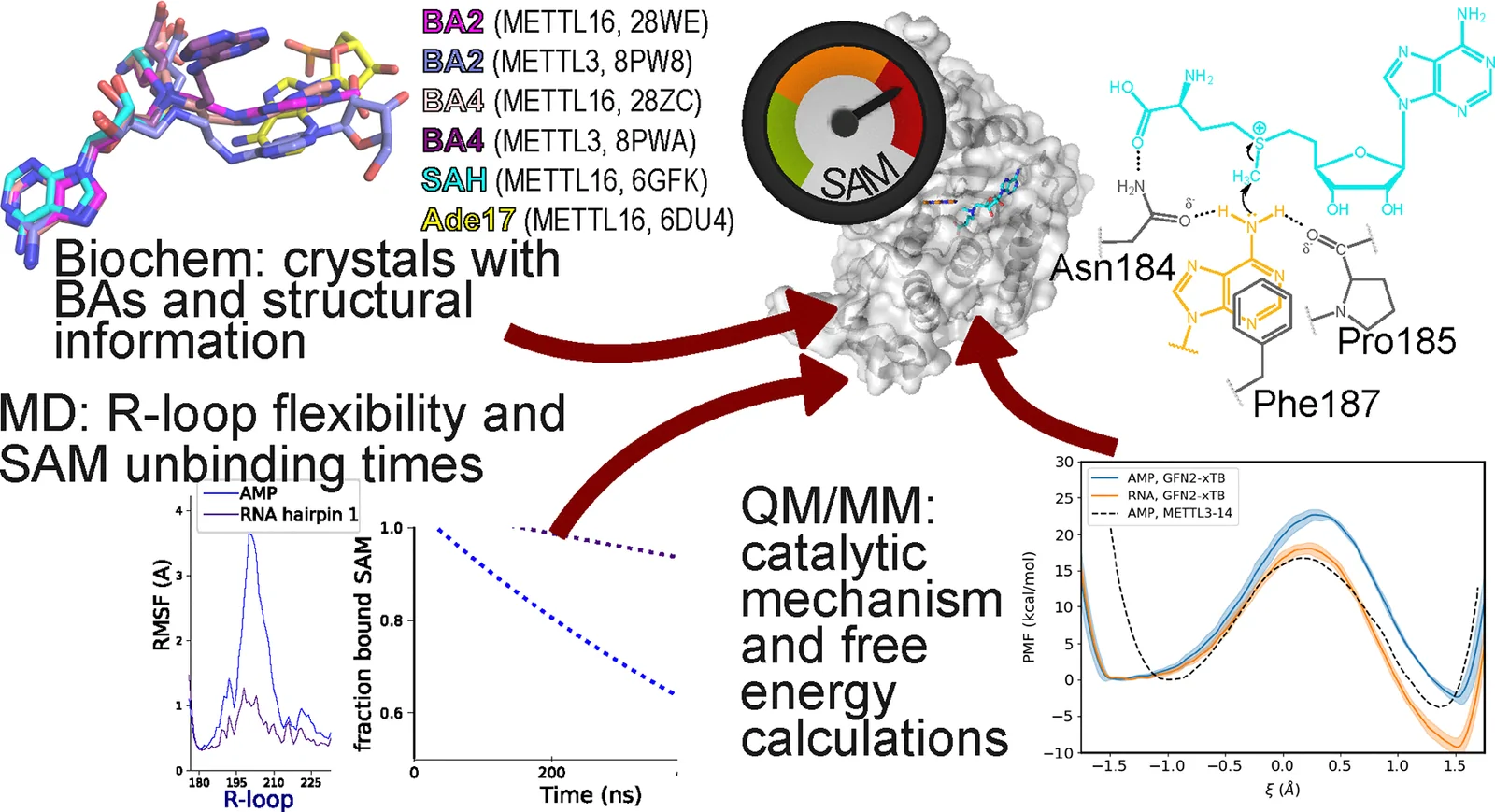

## 1. Introduction

### 1.1. METTL16 catalyzes the adenine-N6 methylation within structured motifs of MAT2A mRNA and U6 snRNA

Similar to how epigenetics deals with chromatin modifications and their influence on gene transcription, the field of epitranscriptomics studies the modifications of RNA and how they modulate the fate of the RNA transcripts. More than 170 RNA modifications have been identified,^1^ and on mRNA, the most frequent modification is N6-methyladenosine (m6A).^2^ m6A is involved in most aspects of RNA regulation, such as alternative polyadenylation,^3^ splicing,^4^ nuclear export,^5^ stability^6^ and translation initiation.^7,8^ Most m6A marks are installed by the complex of methyltransferase-like protein 3 (METTL3) and 14 (METTL14) within the consensus sequence GGACU.^9^ Methyltransferase-like protein 16 (METTL16) is a distinct m6A writer enzyme, for which two RNA substrates have been identified so far.^10,11^ Unlike the METTL3–14 heterodimer acting on linear single-stranded RNA, METTL16 is monomeric and methylates only RNAs with a specific tertiary structure that contain the recognition motif UACA̲GARAA (R = A or G, underlined A gets methylated).^11,12^

The known substrates of METTL16 are U6 spliceosomal RNA and MAT2A mRNA, the latter of which encodes the enzyme responsible for the synthesis of *S*-adenosyl methionine (SAM). The METTL16-catalyzed methylation of the U6 snRNA at position A43 is located in the catalytic unit of the spliceosome and is required for efficient and accurate splicing of specific pre-mRNAs.^11^ METTL16 installs methylation marks at six hairpins in the 3′ UTR of the MAT2A pre-mRNA. These methylation marks play a key role for the regulation of the MAT2A enzyme translation, which controls the SAM levels in the cell.^11,12^ At low SAM levels, METTL16 induces splicing of the final MAT2A intron by binding the unmethylated hairpins without methylating them. This leads to increased MAT2A and SAM production. When SAM levels increase, METTL16 methylates the MAT2A mRNA at the aforementioned hairpins, which leads to intron retention and increased degradation of the MAT2A transcript. Thus, METTL16 can be viewed as an intracellular SAM sensor and is important for maintaining SAM levels in a physiological range.^11,13^ For its function as a SAM sensor, it is critical that the enzymatic activity of METTL16 is tuned to the range of physiological SAM levels. The intracellular concentration of SAM in different mammalian cells ranges from 0.1 µM to 1 mM.^14^ The high Km of METTL16 for SAM, which is in the range of 130–400 µM,^15,16^ is consistent with the high-micromolar intracellular concentration of SAM.

Several crystal structures of the METTL16 methyltransferase domain (MTD) have been solved in the past.^12,17,18^ Among these structures, two show the apo enzyme without co-factor or substrate bound (PDB-ID: 6B91 and 6GT5), four depict METTL16 in complex with *S*-adenosyl homocysteine (SAH, PDB-ID: 2H00, 6B92, 6GFN and 6GFK), and two structures show the enzyme bound to MAT2A RNA hairpin 1 or 6 (PDB-ID: 6DU4 and 6DU5). The structures reveal that the METTL16 MTD adopts a Rossmann-fold, highly conserved in Class I SAM-Methyltransferases (MTases), preceded by a unique N-terminal segment (Fig. 1A). The N-terminal segment contains several positively charged amino acids and is essential for RNA-substrate binding. Another unique feature of the METTL16-MTD structure is the R-loop consisting of residues 189–213 (Fig. 1B, highlighted in blue), which is only resolved in the RNA-bound structures and tightly interacts with the RNA substrate.^12^ This observation suggests an induced-fit mechanism for substrate RNA binding.

**Fig. 1:**
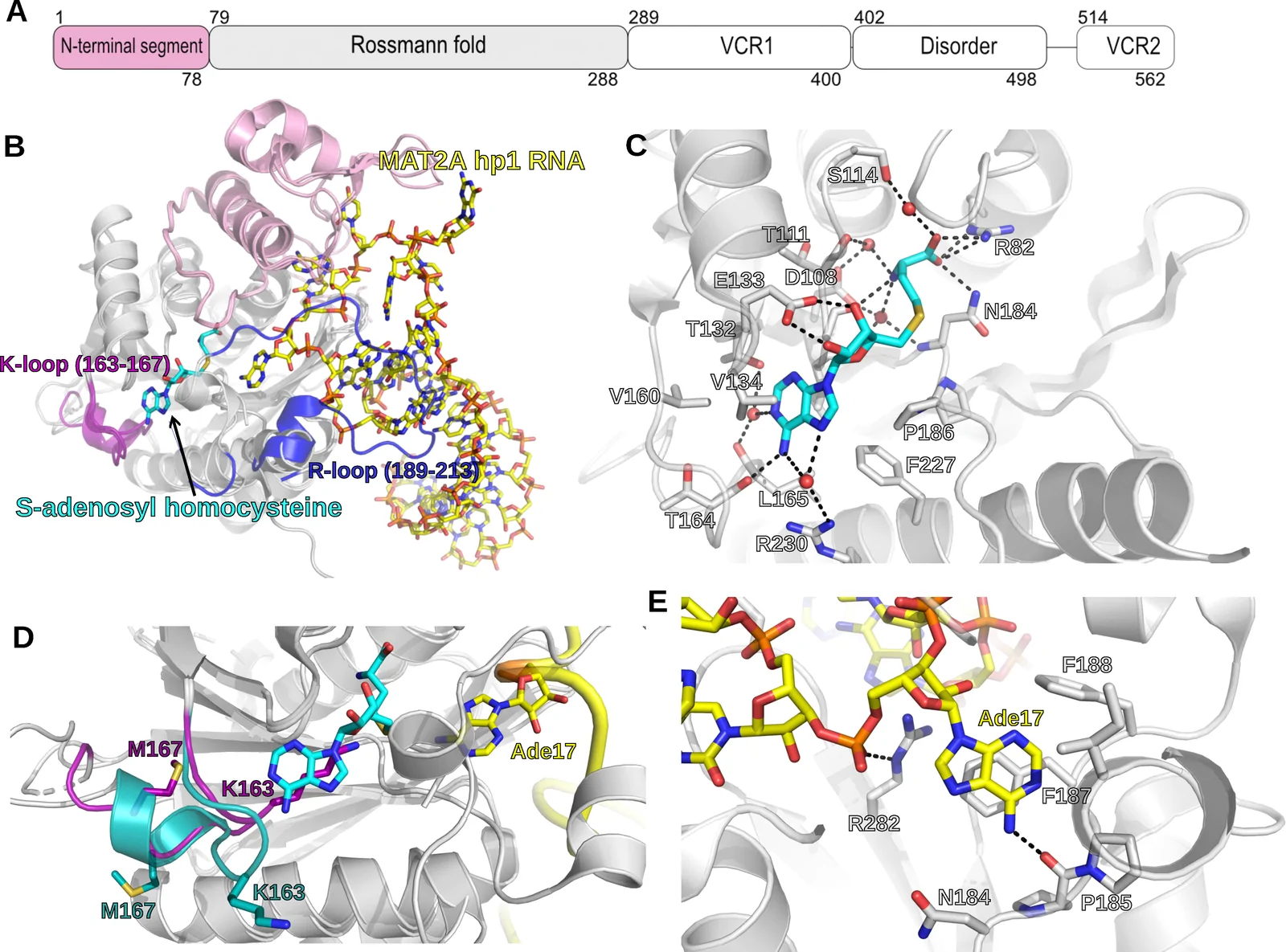
Structural features of the RNA methyltransferase METTL16. (A) Domain architecture of METTL16. VCR = vertebrate conserved region. (B) Overlap of known METTL16 structures (6B91, 6GFK, 6DU4). The structured part of METTL16 consists of its N-terminal segment (residues 1–78, pink) and the Rossmann fold (residues 79–288, gray). The structure of SAH originates from 6GFK (carbon atoms in cyan), and the hairpin 1 (hp1) of MAT2A mRNA from 6DU4 (yellow). The K-loop spans residues 163–167 (purple) and the R-loop residues 189–213 which are only resolved in the structure with RNA (6DU4, blue). (C) Close-up of the SAH binding pose in 6GFK. Polar interactions are marked (dotted lines). (D) Close-up of the different K-loop conformations in the complex with the hairpin 1 (6DU4, loop in purple and RNA in yellow) or SAH (6B92, loop and SAH in cyan). (E) Close-up of RNA Ade17 binding pose in 6DU4.

A catalytic mechanism has been proposed based on the crystal structures. The 184-NPPF-187 motif is central for catalysis.^19^ The acceptor N6 amino group of the substrate adenine is polarized through hydrogen bonds by the Oδ atom of N184 and the backbone carbonyl oxygen of P185. Furthermore, F187 stabilizes the orientation of the substrate adenine through π–π-stacking interactions (Fig. 1E).^12^

Even though structures of METTL16 in complex with a substrate RNA and in complex with SAH are known, no structure of METTL16 bound to both the substrate RNA and cofactor SAH/SAM simultaneously has been solved. Interestingly, the so-called K-loop takes on an alternative conformation in the METTL16-RNA complex, which leads to K163 occupying the cofactor pocket, thus rendering it impossible for SAM to bind (Fig. 1D).^12^ Mutating K163 to alanine increases the methylation activity of METTL16 in biochemical and cellular assays.^12,13,20^ Therefore, the K-loop is believed to enable METTL16 to inhibit its own activity, albeit the exact mechanism of this autoregulation is not fully understood.

### 1.2. Bisubstrate analogs as structural tools to investigate the mechanisms of MTases

To study the molecular mechanism of methyl transfer catalyzed by SAM-dependent MTases, the structure of the enzyme in complex with both the cofactor SAM and the substrate RNA or DNA is required. Since obtaining a structure of MTases in complex with RNA has been difficult for most MTases due to the intrinsic instability of RNA and RNA-enzyme complexes, bisubstrate analogs (BAs) have been designed to study the catalytic mechanism of m6A-MTases.^21–24^ BAs covalently link an acceptor nucleoside to the cofactor SAM and aim to mimic the transition state (Fig. 2). To obtain a stable molecule, the sulfur atom of SAM was replaced by a nitrogen, to which N6 of the substrate adenosine was attached through a linker.

**Fig. 2:**
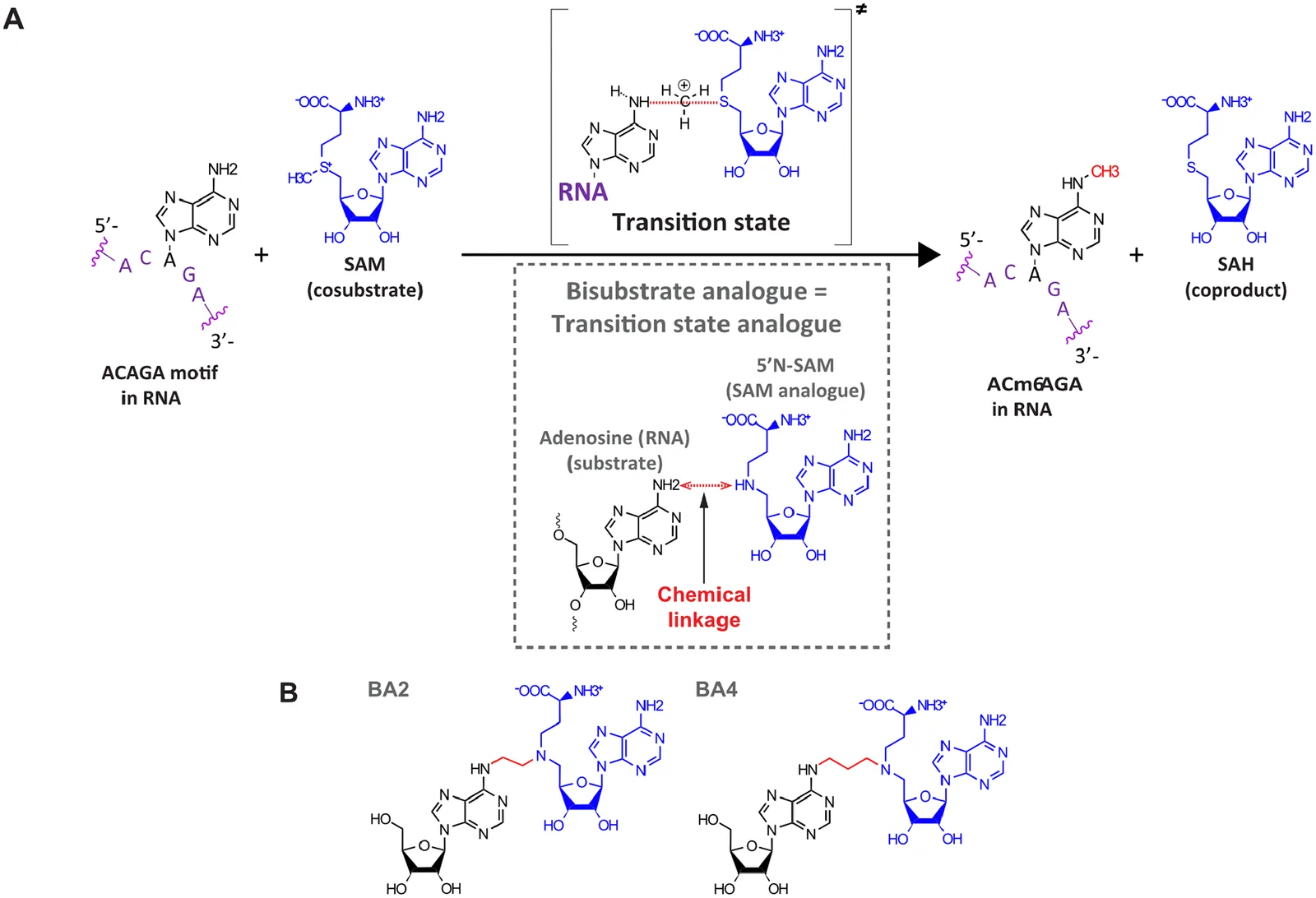
Bisubstrate analogs (BAs) as transition state mimics for METTL16. (A) Methyltransfer reaction catalyzed by METTL16. The methyl-group of the co-substrate SAM (blue) is transferred to position N6 of adenine (black) within the ACAGA motif. The transition state of the reaction is depicted above the horizontal arrow and the design principle of BAs as transition state mimics below the arrow (dashed box). (B) Chemical structures of the BAs used in this study. BA2 and BA4 have two and three methylene groups in the linker (red), respectively.

Structures of RNA m6A MTases in complex with BAs have helped understand the catalytic mechanism of the bacterial enzyme RlmJ^24^ and human METTL3–14.^25^ The complex of METTL3–14 and BA2 represents a transition state binding conformation, whereas the BA4 structure shows an intermediate of RNA binding. Molecular dynamics (MD) simulations starting from the complexes of METTL3–14 and BA2 or BA4 revealed the role of METTL3 Y406 in capturing and guiding the substrate nucleoside to the catalytic site *via* π–π-stacking interactions. Furthermore, hybrid QM/MM free energy simulations carried out using the structure of METTL3–14 with BA2 proposed a mechanism of methyl transfer without prior deprotonation of the adenosine.

Here, we aimed to solve crystal structures of METTL16-MTD in complex with BA2 and BA4. These structures were then used as starting point for MD and QM/MM simulations with the objective to elucidate the molecular mechanism of methyl transfer catalysis by METTL16.

## 2. Results and discussion

### 2.1. Bisubstrate analogs mimic the transition state

We obtained crystal structures of METTL16-MTD in complex with bisubstrate analogs BA2 (28WE) and BA4 (28ZC) at resolutions of 2.3 and 2.2 Å, respectively (Fig. 3A and B). The SAM-moiety of the BAs aligns well with SAH from previously reported structures (6GFN, 6GFK, 6B92, 2H00). Analogous to SAH, the adenine of the BA's SAM-moieties is located in a hydrophobic cage formed by I109, V134, V160, L165 and F227. The adenine-N6 atom of the BA's SAM moiety forms a hydrogen bond with the backbone carbonyl of T164. Adenine N1 is involved in a water-mediated hydrogen bond with T132. The ribose 2′- and 3′-hydroxyl groups interact with the conserved E133 and the carboxylate of the SAM-methionine moiety forms a salt-bridge with R82. The α-amino group of SAM forms a direct hydrogen bond with G110. A network of hydrogen bonds involving three waters and residues D108, G110, T111, S114 and Y117 further stabilizes binding of the SAM-methionine moiety.

**Fig. 3:**
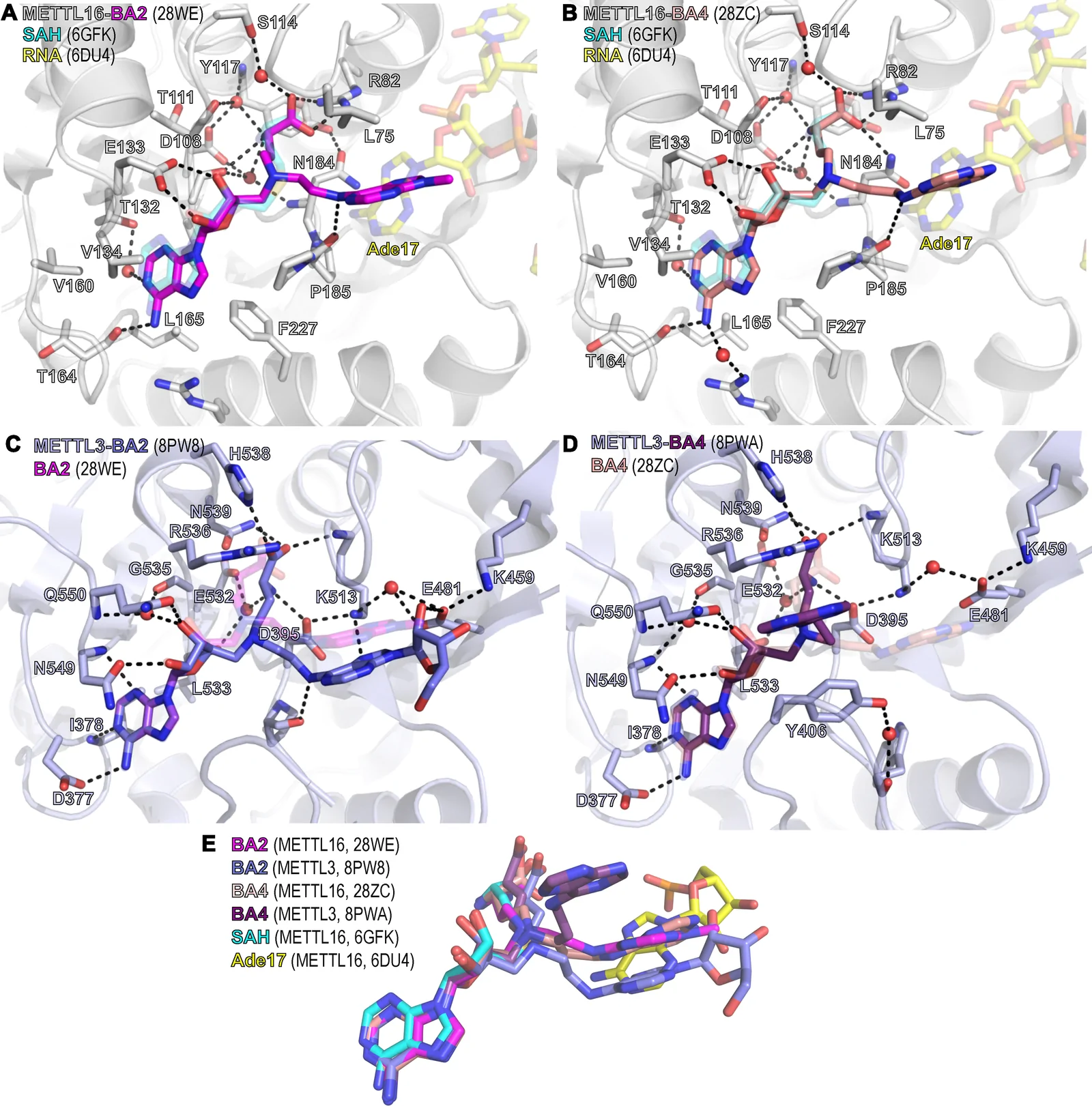
Crystal structures of METTL16-MTD in complex with bisubstrate analogs (BAs) and comparison with METTL3. (A) The METTL16 complexes with BA2 (PDB 28WE, carbon atoms in magenta) and (B) with BA4 (PDB 28ZC, salmon) are superimposed to the METTL16 complexes with SAH (transparent cyan) and RNA (transparent yellow). (C) METTL3/BA2 structure (METTL3 in gray, BA2 in slate) superposed on METTL16/BA2 (BA2 in transparent magenta). (D) METTL3/BA4 structure (METTL3 in gray, BA4 in purple) superposed on METTL16/BA4 (BA4 in transparent salmon). (E) Overlap of BA2 (magenta) and BA4 (salmon) in METTL16 and METTL3, METTL16/SAH (cyan) and METTL16/RNA Ade17 (yellow). In all panels, the ribose ring of BA2 and BA4 in the complexes with METTL16 is not shown because of lack of electron density.

In the NPPF-motif, the N184 side chain orientation optimal for catalysis results in a hydrogen bond between its Nδ atom and the carboxylate of the methionine moiety of SAM/SAH (6GFK, 2H00). This interaction leads to an N184 orientation that enables a hydrogen bond between the N184 Oδ and the acceptor adenine N6 atom. This conformation of N184 has previously only been observed in crystal structures where SAH is bound to METTL16 and not in the apo- or RNA-bound structures, suggesting that SAM-binding stabilizes the N184 conformation for catalysis. The side chain of N184 assumes two different conformations in the two BA structures (Fig. 3A and B). Only in the complex with BA4 is N184 in the active conformation. In the BA2 structure, N184-Oδ forms a hydrogen bond with Y117 instead.

The substrate adenosine moiety of the BAs exhibits a similar conformation as Ade17 in the structure of METTL16 bound to the hairpin 1 of MAT2A (6DU4). The substrate adenine N6 atoms of BA2 and BA4 are 2.1 and 2.3 Å away, respectively, from the corresponding atom in the RNA substrate upon superposition with the 6DU4 structure. Moreover, the distance between adenine N6 and the P185 carbonyl oxygen is 3.3 and 4.0 Å in the BA2 and BA4 structure, respectively. This is close to the distance required for the formation of a hydrogen bond. Furthermore, both structures reveal a hydrophobic contact between the RNA-mimicking adenine of the BAs and the side chain L75. Similar to the available apo and SAH-bound structures of METTL16, there was no electron density for the region spanning residues 187–215. This is consistent with the observation that the R-loop (residues 189–213) only becomes structured upon substrate RNA binding. The RNA mimicking adenosine of the BAs is too small to trigger the ordering of the R-loop, since the structural feature of the RNA substrate seems to be most important for the recognition by METTL16.

It is interesting to compare the METTL16/BAs complexes and the corresponding complexes with METTL3–14 which is the main m6A-RNA writer enzyme. The structural superposition shows that the conformation of both BA2 and BA4 in METTL16 resembles BA2 in METTL3 (Fig. 3C and D). The structure of METTL3 with BA2 mimics a possible transition state of methyl transfer and BA4 is likely an intermediate of RNA substrate binding.^25^ In the case of METTL16, both complexes with BA2 and BA4 mimic the putative transition state. This is also supported by the angles and distances between the SAM-CH3 group and the acceptor adenosine N6 (see Fig. 4). The optimal angle between 5S, Cε of SAM and N6 of adenine for an SN2 reaction is 180°. Indeed, in the BA2 and BA4 structures, this angle is very close to the optimal value (141° and 174°, respectively) and the donor methyl and acceptor N6 groups are very close (2.5 and 2.8 Å, respectively).

**Fig. 4:**
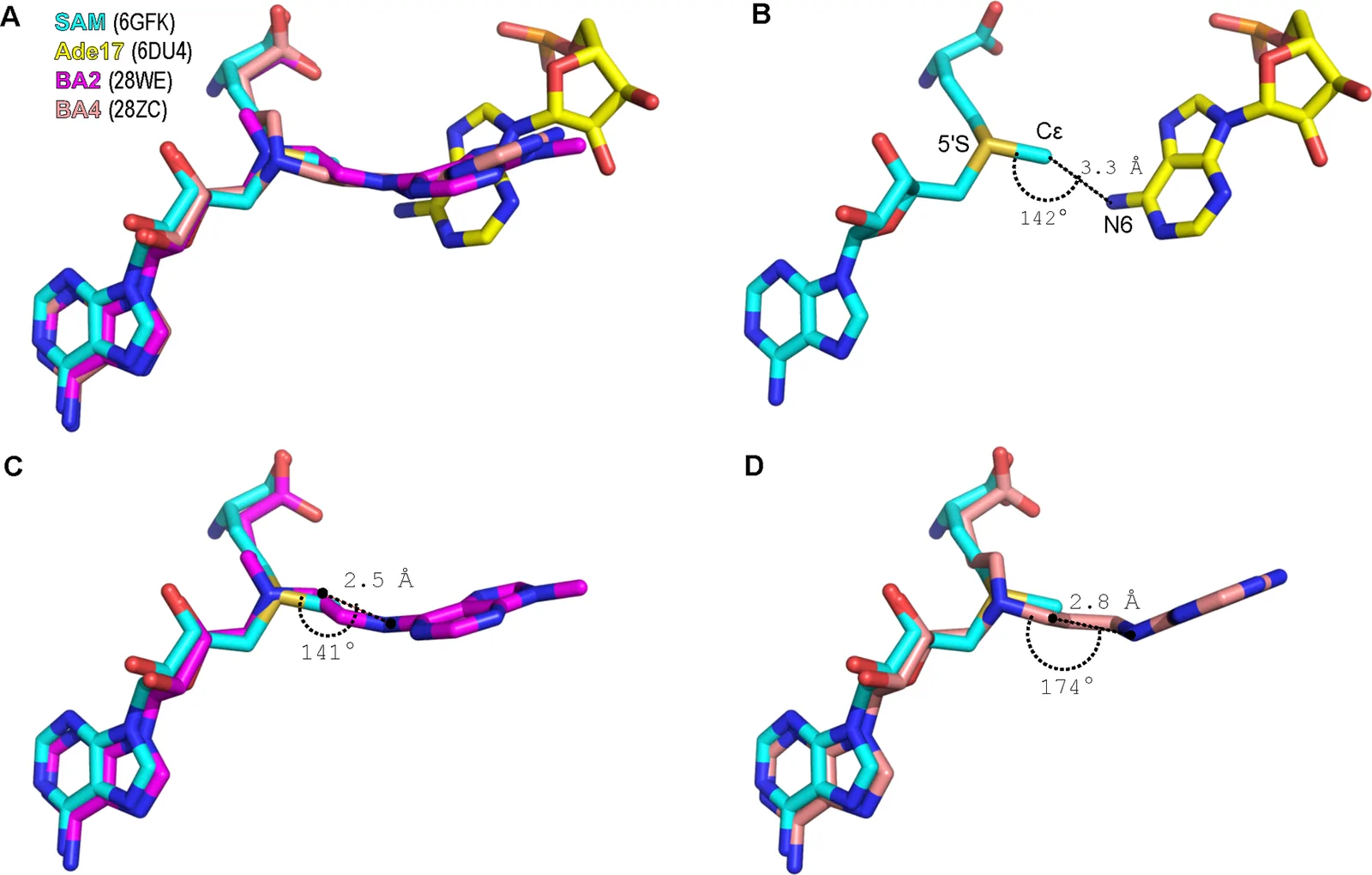
Analysis of the BA2 and BA4 conformations in the crystal structures of the complexes with METTL16. (A) Superposition of SAM from 6GFK (cyan, the Cε group was added to SAH using the Chem3D software), Ade17 from 6DU4 (yellow), BA2 from 28WE (magenta) and BA4 from 28ZC (salmon). (B–D) The distance between SAM-Cε and Ade-N6 and the angle SAM-5S-Cε-Ade-N6 are shown. In all panels, the ribose ring of BA2 and BA4 in the complexes with METTL16 is not shown because of lack of electron density.

The structural alignment of the active sites of METTL3 and METTL16 reveals similarities and differences between these two RNA MTases. The catalytic NPPF-motif in METTL16 is analogous to DPPW in METTL3. Our previous study of the methyl transfer catalyzed by METTL3 showed that D395 and P396 play a central role in stabilizing the transition state. In the structure of METTL3 bound to BA2 (Fig. 3C), D395 forms a salt bridge to the SAM-methionine moiety of BA2 and a hydrogen bond to N6 of the substrate-adenine moiety. Furthermore, a very stable salt bridge between D395 and K513 is formed, stabilizing the D395 conformation. In METTL16, the equivalent of D395 is the uncharged N184, which exhibits more disorder than D395 in METTL3, as it can be observed in different conformations among the various METTL16 crystal structures. In the active state, the conformation seems to be stabilized by cofactor binding through a hydrogen bond between SAM and the Nδ atom of N184. While K513 stabilizes the D395 rotamer for catalysis in METTL3, no polar residues in the vicinity of N184 fulfill that function in METTL16. Another difference between the METTL3 and METTL16 active sites is the extent of the polar interaction network stabilizing interactions with both SAM and the substrate. In METTL3, a network of six consecutive salt bridges was detected throughout the catalytic core. These ionic interactions are less pronounced in the METTL16 active site. This difference in electrostatic properties of the catalytic pocket could lead to different reaction profiles of the two methyltransferases. A substantial difference between the two methyltransferases is observed in their interaction with the N1 of the SAM adenosine. In METTL3 there is a direct hydrogen bond between the backbone amide of I378 and N1, while METTL16 shows a water mediated hydrogen bond between N1 and the side chain of T132 (Fig. 3). This difference explains, at least in part, the lower SAM affinity of METTL16, which is essential to its function.

To further investigate the methyl transfer reaction catalyzed by METTL16, we conducted classical MD and QM/MM free energy simulations using the BA2 and BA4 crystal structures as the starting point.

### 2.2. MD simulations confirm loose binding of the cofactor SAM

We used atomistic MD simulations to study the stability of the bound state of (co-)substrates and (co-)products. Multiple independent simulations were carried out for each METTL16 complex with RNA substrates of different lengths. The calculated unbinding times are shown in Table 1 and Fig. S1. The binding of the co-substrate/co-product was monitored using the average value of five distances between individual groups of SAM/SAH and METTL16 (see Section 3.5 and Fig. S2–S7). These distances were chosen because of their relevance in the crystal structures (Fig. 3) and monitored throughout the simulation. The hydrogen bond between the backbone carbonyl of T164 and N6, which stabilizes the adenine moiety in the crystals, becomes water-mediated in most simulations. The side chain of E133 stabilizes the ribose hydroxyl groups *via* hydrogen bonds. The salt bridge between R82 and the SAM carboxyl group stabilizes the methionine moiety. Furthermore, we monitored the hydrogen bond between the N184:ND2 and the carboxyl group, which has been shown to be necessary for catalysis.

**Table 1:** Unbinding times of substrate/product and SAM/SAH from METTL16

METTL16 with	Unbinding times (ns)	
	Substrate/product	SAM/SAH
CAGA/SAM	1162	970
Cm6AGA/SAH	567	2603
CAGA/—	338	—
AMP/SAM	388	774
m6AMP/SAH	90	≫1000
F187A AMP/SAM	113	≫1000
R282A AMP/SAM	15	≫1000

The binding of the substrate/product was defined by a distance between P185:O and N6 of the adenine shorter than 7.5 Å (See Fig. S8 and S9). In the simulations started from the complex with the MAT2A hairpin 1 RNA, no unbinding of the RNA substrate was observed. Thus, we also conducted MD simulations of METTL16 in the complex with the tetranucleotide RNA motif (CAGA and Cm6AGA) and only AMP and m6AMP, which are much smaller than the hairpin-RNA. The weaker conformational constraints of the shorter oligonucleotide substrates or products result in an increased flexibility of METTL16 and unbinding of the substrate or product within timescales of about 1 µs. Dissociation is faster for the mononucleotides than tetranucleotides, and for the methylated products than the unmethylated substrates (Table 1, Fig. S1). Interestingly, the CAGA substrate in absence of SAM shows similar adenosine unbinding rates as AMP with SAM, which provides evidence for the importance of SAM in the stabilization of the RNA substrate. The significantly higher stability of the bound state of the hairpin 1-RNA substrate is consistent with the experimental data on METTL16 substrates, which are RNAs with stable secondary structure.^10,16,26,27^

We then carried out MD simulations with the METTL16 mutants F187A and R282A which are catalytically impaired. These simulations show fast unbinding of the AMP substrate (Table 1). They provide further evidence of the role of the π-stacking between F187 and the adenine as an essential interaction for binding and catalysis, and that of R282 to clamp the RNA backbone phosphate. This role was also shown by mutation studies in which replacements of these residues resulted in more than twice weaker RNA binding than the wild-type.^20^ The mutation of F187G resulted in an almost 300-fold loss of catalytic efficiency.^20^

The unbinding of SAM from METTL16 was observed within a sub-microsecond time scale in the presence of AMP or CAGA. The rapid unbinding of SAM is in contrast to our previous METTL3–14 simulation study in which we did not observe SAM unbinding events.^25^ This is not unexpected, given the three orders of magnitude lower affinity of SAM for METTL16 than METTL3,^16^ and the role of METTL16 as a SAM concentration sensor. The greater structural stability of the SAH binding mode resulted in slower unbinding than SAM (Fig. S2–S7). It is interesting to note that the interactions between METTL16 and the adenine ring of both SAM and SAH are weak. In contrast, the contacts with the ribose and methionine moiety are significantly more stable for SAH than SAM. This difference is due, at least in part, to electrostatic repulsion between the positively charged sulfonium cation of SAM and the basic side chains of METTL16.

### 2.3. The R-loop flexibility decreases in the presence of RNA

For most of the simulation systems, the greatest displacements are observed in the N-terminal segment (residues 1–78) and the R-loop (residues 189–213, Fig. 5 and Fig. S10–S12) of METTL16. These flexible segments are substantially stabilized by the hairpin 1 RNA and not by smaller RNA substrates. In contrast, the 10-residue loop G94-L103 and K-loop show the same plasticity in all simulations. The positively charged residues within the RNA clamp (R74, R279, and R282) exhibit minimal flexibility upon binding the hairpin RNA, which agrees with their functional roles in RNA anchoring and stabilization. Similarly, the N-terminal segment is also stabilized, even in regions that do not interact with RNA directly.

**Fig. 5:**
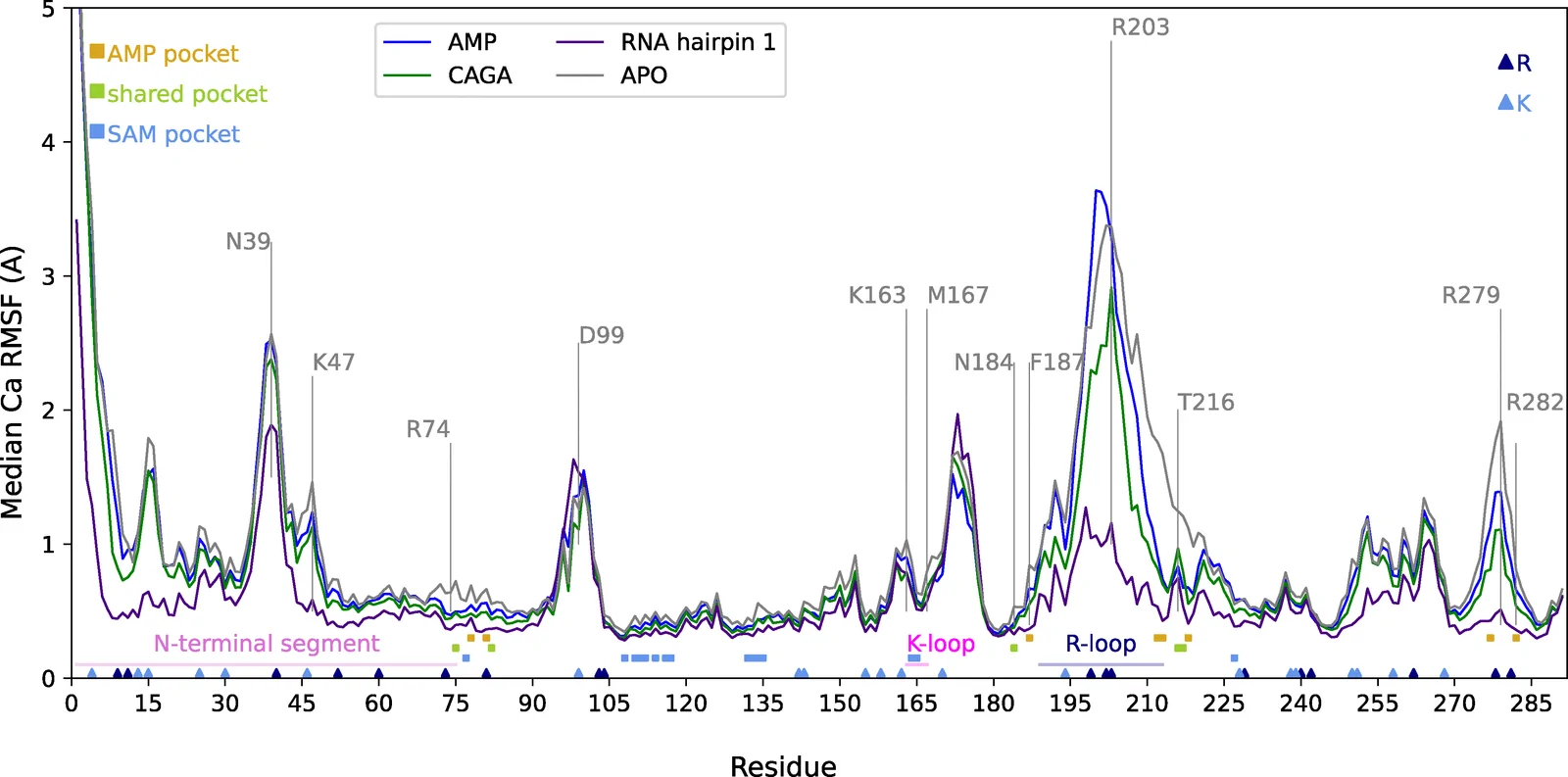
Median Cα root-mean squared fluctuation (RMSF) profiles of METTL16 complexes with substrate (AMP, CAGA, RNA) and apo state. The RMSF decreases, *i.e.*, the structural stability increases, for larger RNA substrates (RMSF of AMP > CAGA > RNA hairpin 1). Charged residues are marked as blue triangles (dark: R, light: K). Relevant structural regions of METTL16 are labeled (horizontal segments at the bottom), and the binding pockets are emphasized (squares at the bottom with legend at the top left). Residues mentioned in the text and/or relevant for RNA binding are explicitly labeled.

As mentioned above, the R-loop shows large displacements in all MD simulations except for the MD runs of the complex with the RNA-hairpin (Fig. 5). The distance between the tip of the loop (R203) and the RNA-adenosine pocket (R282) fluctuates between 10 and 50 Å (Fig. 6 and Fig. S14). The flexibility of charged segments of METTL16 in the absence of RNA is reminiscent of the recognition mechanism observed in atomistic simulations of the binding of acetylated histone tails by the ATAD2 bromodomain.^28^ This mechanism was described as “fly-casting” due to the recruitment of the charged ligand by electrostatic complementarity and a flexible loop of the bromodomain (called ZA loop residues D1003–D1030). The length of the METTL16 R-loop (25 residues) is similar to the ZA loop of the ATAD2 bromodomain (28 residues). However, the METTL16 R-loop has three arginine side chains since the RNA substrate is negatively charged, while the ATAD2 ZA loop contains acidic side chains since histone tails are positively charged. Originally, fly-casting was proposed as a mechanism for kinetic acceleration (*i.e.* by allowing a larger capture radius) of the binding of partially disordered proteins to DNA, with folding happening after binding.^29^ Further research showed that apart from larger capture radii, which is offset by slower diffusion, disordered regions have lower energetic barriers for binding their partners. This, and not the larger radius, was proposed to be the main advantage of these regions.^30^ Fly-casting, or mechanisms based on the same induced folding principle, are extended in fuzzy protein–DNA interactions,^31^ disordered proteins,^32^ or protein–peptide.^33^ Several pieces of evidence support the fly-casting mechanism in METTL16: (i) the lack of electron density in the absence of the RNA hairpin, (ii) the extreme flexibility of the R-loop even with the tetrameric RNA (Fig. 7), and (iii) the large capture radius of the arginines in the R-loop (Fig. 8). It is likely that the fly-casting mechanism is more general, *i.e.*, other proteins make use of a flexible loop for recruiting charged (poly)peptides or (single-stranded) nucleic acids by electrostatic complementarity as observed in our simulations of two completely different proteins involved in epigenetics (ATAD2 bromodomain) and epitranscriptomics (METTL16), respectively.

**Fig. 6:**
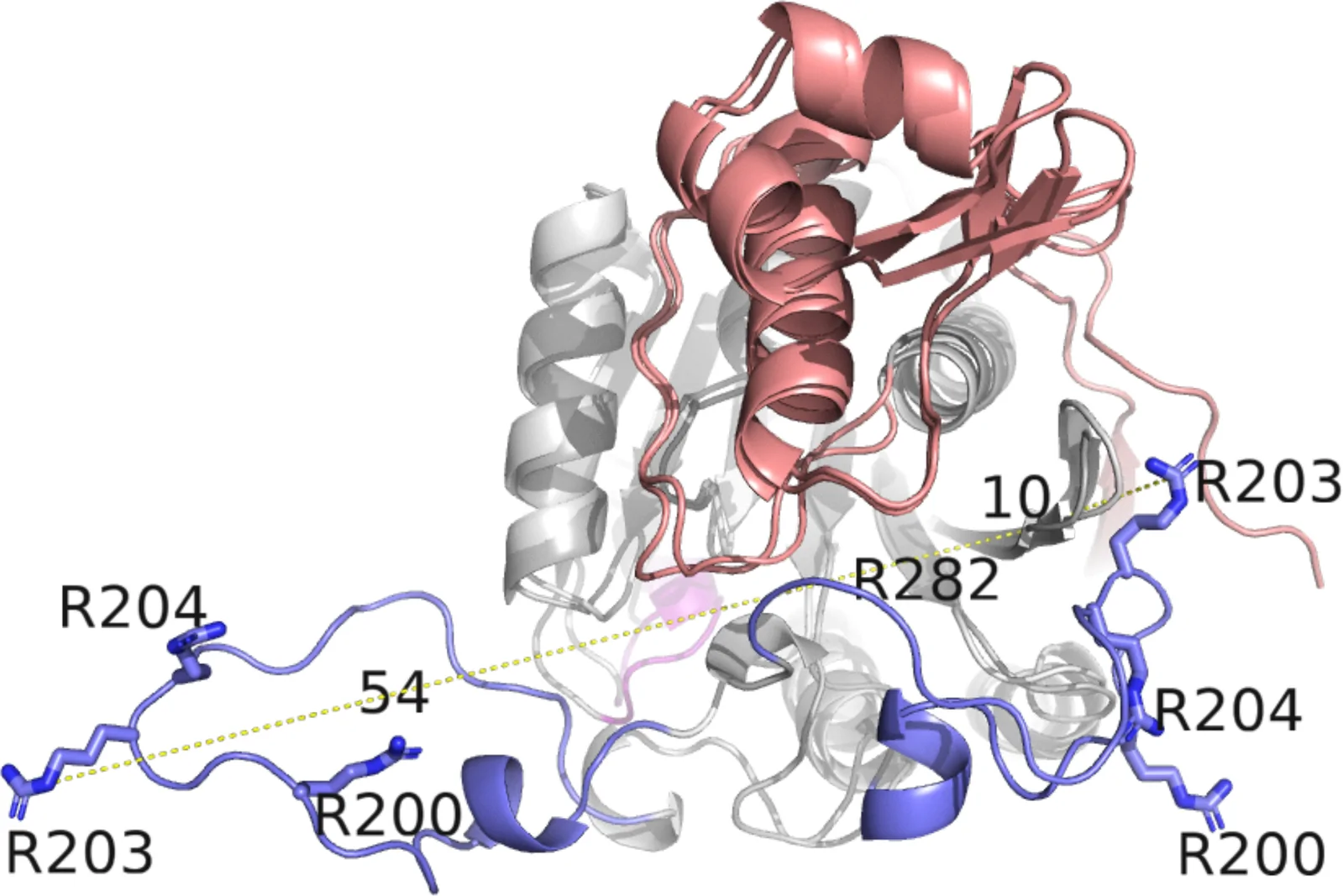
Intrinsic flexibility of the METTL16 R-loop (residues 189–213). Two representative snapshots from the apo simulations are shown to illustrate the distance between the tip of the loop, *i.e.*, the R203 guanidinium group, and the Cα atom of R282 in the active site (10 Å and 54 Å, respectively, color analogous to Fig. 1B). The basic side chains R200, R203, and R204 are emphasized (sticks). The flexibility of the R-loop facilitates the recruitment of RNA by the “fly-casting” mechanism.

**Fig. 7:**
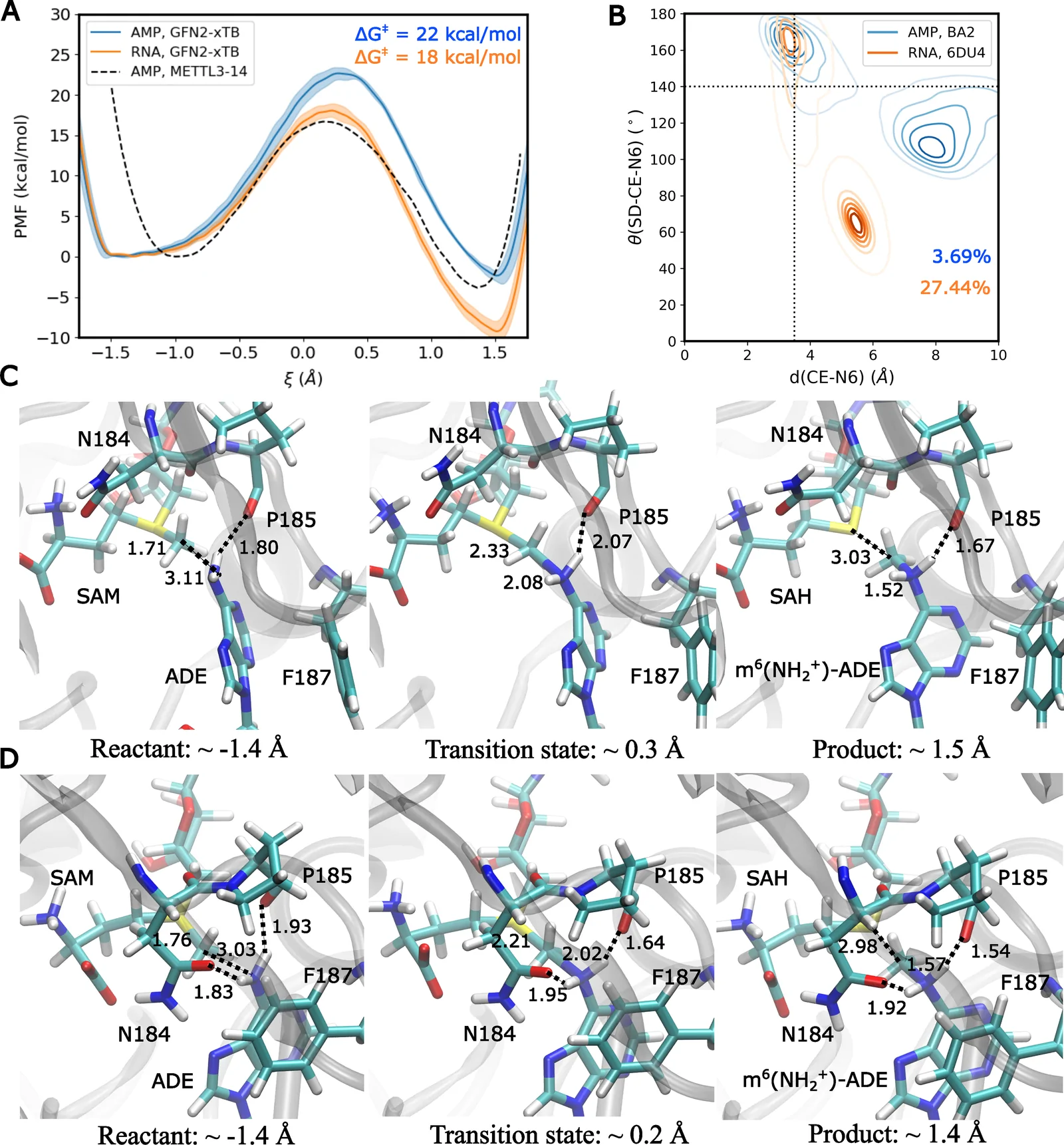
Reaction mechanism of the methyl transfer catalyzed by METTL16 from QM(GFN2-xTB)/MM simulations. (A) Potential of mean force (PMF) along the antisymmetric stretch coordinate *ξ* = *r*(CE–SD) − *r*(CE–N6) for the SAM-AMP system (AMP) and the SAM-RNA hairpin 1 system (RNA). The solid curve and shaded area denote the mean and standard error from three independent runs, respectively. The PMF of METTL3–14 is plotted as a basis of comparison. (B) Nucleophile attack distance-angle distribution from classical MD simulations. The contour lines enclose 90% of the total distribution. The percentage of frames with a distance <3.5 Å and an angle >140° is shown in the lower right. (C) and (D) Active site snapshots of the reactant, transition state, and product for the (C) SAM-AMP and (D) SAM-RNA hairpin 1 complexes. Key interatomic distances (in Å) between reactive groups (SAM and ADE) and proximal residues (N184–F187) are indicated; the METTL16 backbone is displayed in gray ribbon representation.

**Fig. 8:**
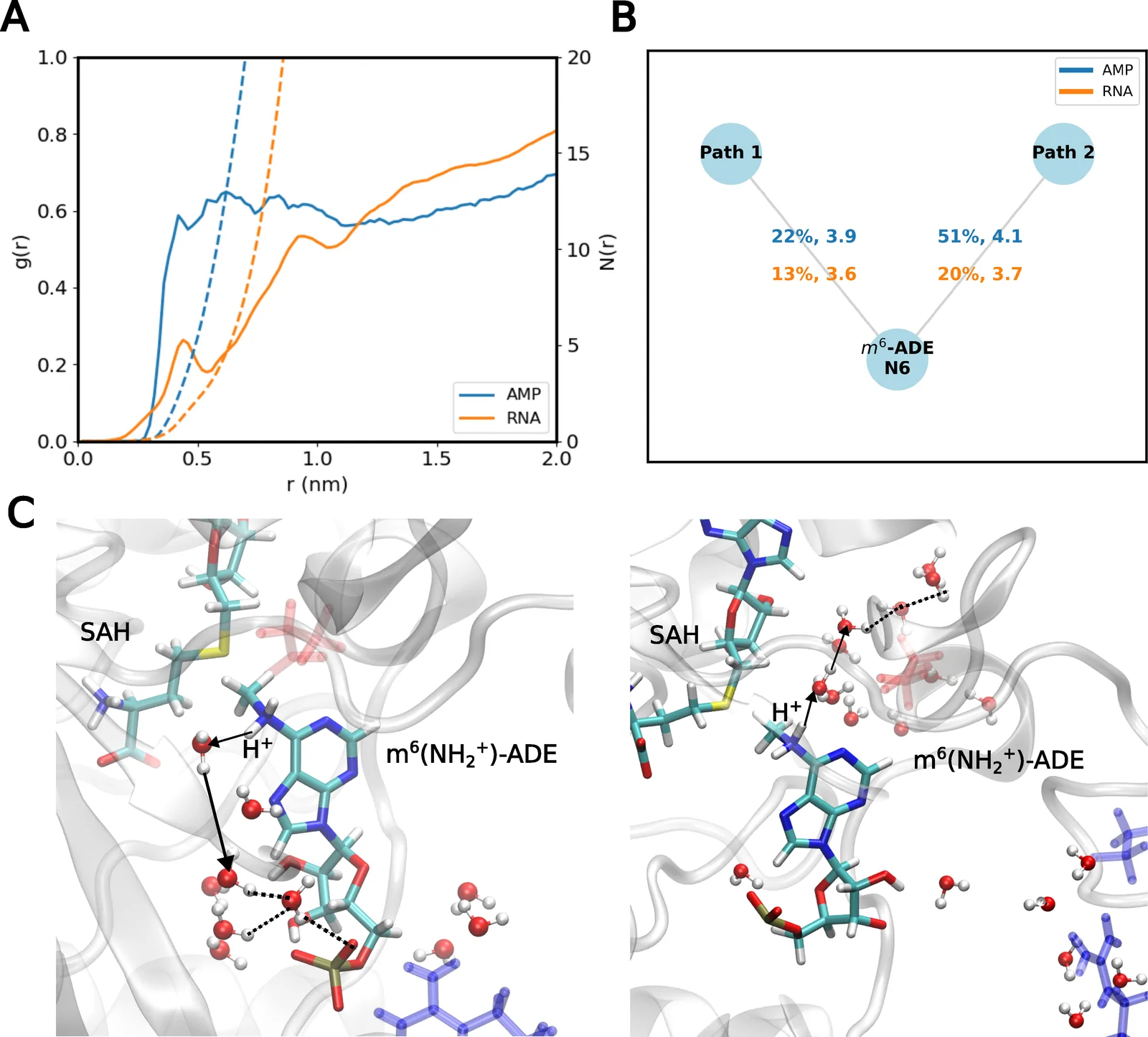
Water-wire mediated mechanism for the deprotonation of m^6^(NH_2_^+^)-adenosine in METTL16. (A) Radial distribution function (RDF) and coordination number (in solid/dashed lines) of water around m^6^(NH_2_^+^)-adenosine. (B) Average occupancy (in %) and water molecule count within the water wire extending from the product N6 position to the protein–solvent interface along the two paths. (C) Snapshots of the product state from QM/MM simulations of the AMP (left)/RNA (right) systems. METTL16 backbone is shown in gray ribbon representation. Representative water wires and hydrogen bond network observed along path 1 in the METTL16/AMP complex and along path 2 in the METTL16/RNA hairpin 1 complex are highlighted with arrows and dashed line, respectively. Terminal residues for path 1 and path 2 are highlighted in blue and red, respectively.

### 2.4. QM-MM simulations reveal the mechanism of methyl transfer

We carried out hybrid QM/MM^34–36^ free energy simulations to study the methyl transfer reaction mechanism catalyzed by METTL16. Here, the methyl transfer reaction is characterized by the direct transfer of the methyl cation from the SAM cofactor to the adenosine substrate's N6 position, preceding deprotonation at the N6 position. This mechanism is supported by the high p*K*_a_ (∼17) of the substrate adenosine N6-amino group^37^ and the absence of a strong catalytic base near the active site, which contains only polar moieties such as the N184 side chain and the P185/P186 backbone carbonyls. Furthermore, this weakly polar environment is unlikely to induce the significant p*K*_a_ shifts required for deprotonation, while the local hydrogen-bonding network explicitly stabilizes the N6 position over N1, rendering alternative mechanisms involving deprotonation or tautomerization energetically unfavorable. The QM(GFN2-xTB^38^)/MM free energy simulations yielded methyl transfer reaction barriers of approximately 22 and 18 kcal mol^−1^ for AMP and RNA hairpin 1 substrates, respectively (Fig. 7A). These values align with the experimental methyl transfer rate for METTL16 (*k*_chem_ = 0.56 min^−1^),^16^ which corresponds to an activation barrier of ∼20 kcal mol^−1^. Given the lower overall catalytic turnover of METTL16 (*k*_cat_ = 0.07 min^−1^), our QM/MM simulation results suggest that SAH and product RNA dissociation is the rate-limiting step, consistent with findings for the METTL3–14 complex.^25^

The nucleophile attack distance-angle distribution, calculated from classical MD trajectories (Fig. 7B), reveals that the adenine moiety in the RNA-bound complex remains significantly closer to the SAM methyl group than in the AMP-bound system. Specifically, the maximum distance in the RNA-bound complex is ∼6 Å, whereas it extends beyond 10 Å in the AMP-bound system. This narrower spatial distribution indicates tighter substrate binding in the presence of RNA backbone. We also note that the RNA hairpin 1 substrate exhibits a significantly lower activation barrier than that of AMP, likely due to enhanced active site stabilization. In the AMP-bound complex (Fig. 7C), the substrate remains relatively flexible in the catalytic pocket, anchored primarily by a single interaction between the P185 backbone carbonyl and the adenosine amino group. Conversely, the RNA hairpin 1 substrate is stabilized by persistent polar interactions with both the N184 side chain and the P185 backbone carbonyl (Fig. 7D). Furthermore, interactions between the RNA backbone and the METTL16 R-loop promote a conformation that optimizes π–π stacking with the F187 side chain (Fig. 7D). These combined interactions pre-organize the substrate into a transition-state-favorable configuration, thereby reducing the reaction barrier. In addition, this π-stacking not only contributes to lowering the reaction barrier but also stabilizes the positively charged product m6AMP through enhanced charge delocalization and polarization, ultimately yielding a lower product state free energy (Fig. 7A) compared to the AMP-bound system.

Comparison of the free energy profiles of the methyl transfer reaction in the METTL3–14 and METTL16 complexes reveals two primary differences: the activation barrier and the reactant state conformation. METTL3–14 exhibits a lower activation barrier (∼16 kcal mol^−1^) compared to METTL16 (∼22 kcal mol^−1^). Additionally, METTL16 features a more extended reactant state, characterized by a larger distance between the SAM cofactor and the substrate adenosine. This is reflected in an antisymmetric stretch value of −1.5 Å for METTL16 *versus* −1.0 Å for METTL3–14.

These energetic and structural differences stem from distinct interactions within their respective active sites. Although the catalytic motifs 395-DPPW-398 in METTL3–14 and 184-NPPF-187 in METTL16 share sequence similarity, including a central diproline core and a terminal aromatic residue, their local interaction strengths differ significantly. Both complexes maintain a conserved polar interaction between the first proline (P396/P185) and the adenine N6 position. However, P397 in METTL3–14 forms hydrophobic contacts with SAM that are absent for the homologous P186 in METTL16.^25^ Substrate adenine stabilization also diverges between the two enzymes. In METTL3–14, W398 indirectly stabilizes the adenine ring *via* a water-mediated hydrogen bond between its backbone carbonyl and the Y406 side chain, orienting Y406 for π–π stacking with the adenine ring.^25^ Conversely, F187 in METTL16 engages in direct π–π stacking with the adenine ring (Fig. 7). Furthermore, the negatively charged side chain of D395 in METTL3–14 forms a strong electrostatic interaction with the adenine N6 position and participates in a salt bridge with the positively charged side chain of K513, which forms a hydrogen bond with the adenine N7 position in this conformation.^25^ By contrast, the homologous N184 in METTL16 forms a weaker polar interaction with the N6 position. Collectively, the active site of METTL3–14 features more pronounced electrostatic interactions than METTL16. This leads to a highly compact active site conformation, ultimately lowering the nucleophilic attack distance in the reactant state and reducing the activation barrier.

Finally, we propose a water-wire mediated deprotonation mechanism for the protonated *N*6-methylated adenosine. This mechanism is supported by literature estimates of N6-protonated adenosine derivative p*K*_a_ values ranging from −10 to −3,^39^ indicating relatively high acidity. Also, a close inspection of the product state active site from QM/MM simulations reveals sufficient solvation around the m^6^(NH_2_^+^)-adenosine in both AMP and RNA complexes. The radial distribution function (RDF) of water around the adenosine N6 position exhibits a primary peak at ∼4 Å in both systems (Fig. 8A), indicating a localized water molecule that may facilitate product deprotonation. Coordination number analysis reveals that the AMP system exhibits higher solvation than the RNA system. This difference likely stems from the absence of the RNA backbone, which sterically hinders water access to the active site. Furthermore, both RDF profiles display distinct structural features up to approximately 1 nm before converging to bulk-like behavior. This long-range organization suggests the formation of a water wire extending to the protein–solvent interface, as illustrated by the proton release pathway and hydrogen-bonding networks in Fig. 8B and C. Analysis of the hydrogen bond network^40^ extending from the product N6 position to the protein–solvent interface provides insight into water wire occupancy and length (Fig. 8B). The terminal points of these proton release pathways are defined by solvent-exposed residues. Specifically, path 1 is defined by residues K195, R203, and R204 in the R-loop, while path 2 is defined by residue E217. In both the AMP- and RNA-bound complexes, a water wire consisting of approximately four water molecules on average is sufficient to conduct the proton to the protein–solvent interface. Assuming these two pathways operate independently, the average occupancy of at least one continuous water wire in the AMP-bound system is calculated as 1 − (1 − 0.22) × (1 − 0.51) = 0.62. However, following the same model, the average occupancy of at least one continuous water wire drops from 62% in the AMP-bound system to 30% in the RNA-bound system. This twofold decrease suggests that the RNA backbone sterically shields the active site from the solvent, a finding in good agreement with the local RDF analysis. Collectively, these structured water wires originating near the N6 position provide a feasible pathway for product deprotonation and proton release. Paired with the potential of mean force (PMF) profiles for the methyl transfer reaction, these data qualitatively support the mechanism where product deprotonation facilitated by a water wire occurs after methyl transfer.

### 2.5. MD simulations support QM/MM mechanistic interpretation

Several insights from the QM/MM calculations are consistent with the MD simulations. In both the AMP and m6AMP complexes, the unbinding of the mononucleotide is common and disorganized. The complex with CAGA RNA reaches an adenine pose similar to that observed in the complex with the hairpin 1 RNA system used for the QM/MM calculations (Fig. 9A), which is stabilized by π–π stacking of the F187 side chain to the adenine ring (compare Fig. 9B with Fig. 7D). The exit of adenine from the pocket occurs as a fast rotational displacement from the catalytic pocket (Fig. 9C and Fig. S8 and S9). The concomitant rotation of the F187 side chain (around its *χ*_1_ angle, monitored by the distance between the tip of the F187 side chain and the Cα atom of N184) and unbinding of the adenosine is less clearly observed for the complexes with AMP/m6AMP (Fig. 9D and E) than for those with the CAGA tetranucleotide (Fig. 9F–H).

**Fig. 9:**
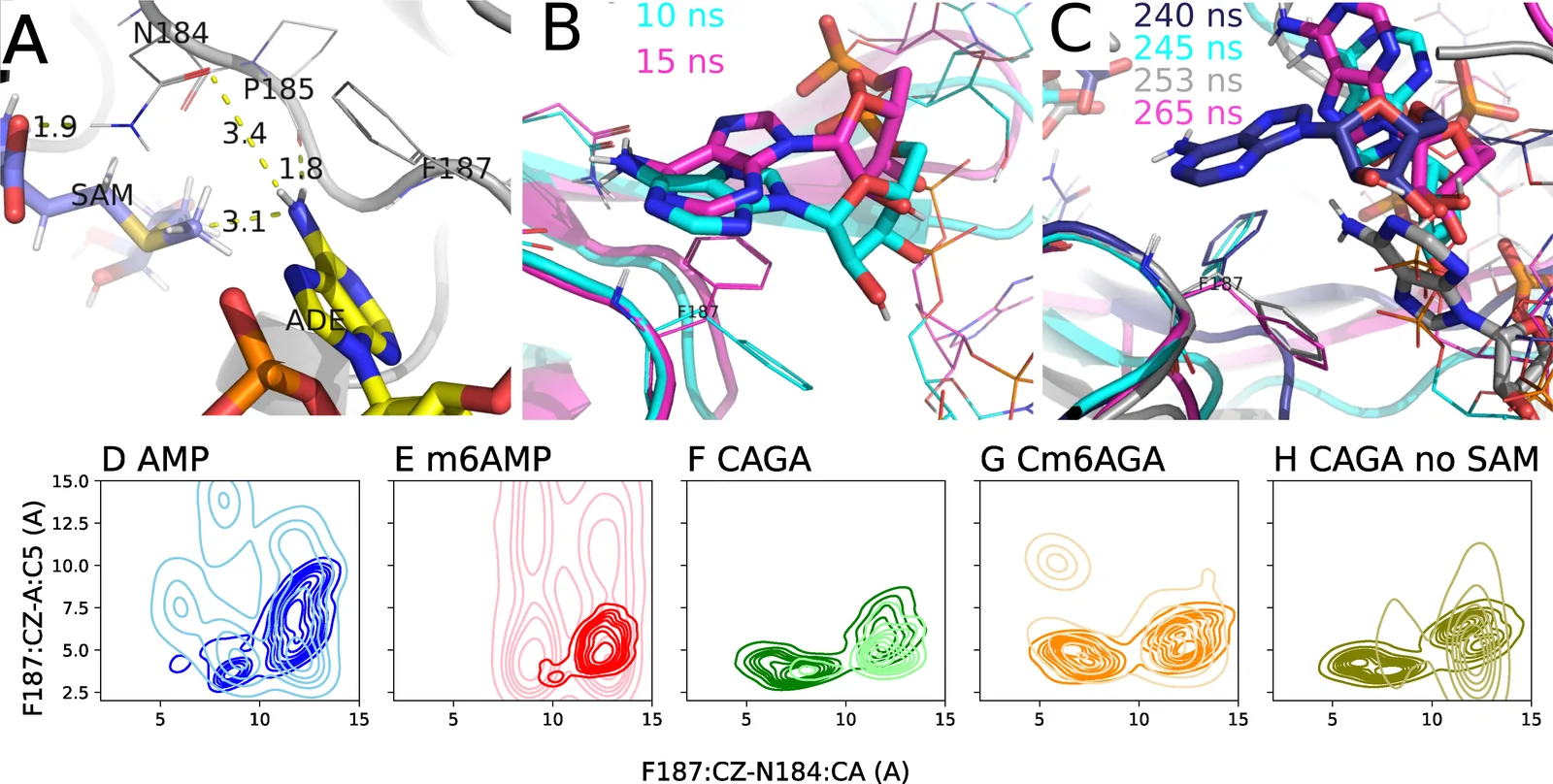
(A) Pose analog to the QM/MM catalytic state, obtained from the CAGA system. (B) The rotation of the F187 side chain occurs very quickly, here exemplified in the interval of 5 ns. (C) Unbinding of CAGA over 25 ns, beginning with the displacement of the adenosine followed by the rotation of the F187 side chain. (D–H) Scatterplot of F187 orientation (distance F187:CZ–N184:CA) *versus* proximity to adenine ring (distance F187:CZ–A:C5). Darker color indicates bound substrate or product, *i.e.*, a distance P185:O–A:N6 < 5 Å.

The rotation of the F187 side chain towards N184 was observed to happen spontaneously and quickly (within 1 ns). The rotational displacement of the adenosine occurs also relatively quickly, followed after less than 10 ns by the rotation of the F187 side chain. In general, the rotation of F187 toward the catalytic pocket (low distance to N184) is observed only in the complexes with the tetranucleotide RNA. In the complexes with the tetranucleotides, the low F187:CZ–N184:CA distance is observed mostly when the adenine is bound. This highlights the role of F187 in the stabilization of the substrate/product in the pocket in presence of structured RNA only. The orientation of the F187 side chain towards the binding pocket was independent of the binding of the substrate/product, but in several cases in the complexes with the tetranucleotide RNA it follows the adenine in its unbinding. In the complex with Cm6AGA (but not with CAGA) unbinding of adenine occurs both with and without rotation of the F187 side chain (Fig. S8 and S9). Examples of the concomitant displacement of the F187 side chain and adenine are observed in trajectories 2 and 7 of CAGA and 5 or 12 of Cm6AGA (Fig. S8). In all cases where a stable rebinding was observed, F187 and the substrate/product remained in close proximity, independently of the orientation of F187 (Fig. S9). The complexes of METTL16 with AMP and m6AMP (Fig. 9D and E) show little involvement of F187 in the stabilization of the adenine ring, meaning that F187 points away from N184 while the adenine is bound, and the unbinding of adenine is independent from the F187 side chain.

As described in the experimental data, and as suggested by the QM/MM simulations, methylation by METTL16 needs not only the stabilization by the binding pocket residues but also by the RNA structure itself. We have observed this also in MD, where the longer RNA chain of the CAGA systems helps constrain the methylated adenine in a position favorable for methylation, meaning low distance between SAM and the substrate, and an angle close to planarity. For the complexes with AMP or m6AMP, there are large fluctuations in the distance and the S–C–N6 angle (S of SAM), even when the adenine is bound (Fig. 10A and B). Practically, this means that a bound AMP can have the right distance to SAM for methylation, but also a very poor orientation (*e.g.*, about 90°). For the CAGA complex and to a lesser extent the complex with Cm6AGA, there are fewer possible conformations of the distance and angle when the adenine is bound. In these cases, a shorter distance is also accompanied by an almost-planar angle, favorable for the proposed reaction mechanism (Fig. 10C and D). In the complex with the CAGA tetranucleotide, the bound adenine adopts the correct angle, approaching 180°, as the distance between SAM:CE–ADE:N6 decreases, and there is no sampling with the SAM–adenine distance shorter than 5 Å and the angle not close to 180°. The complexes with substrate RNA hairpin 1 and product m6A-RNA hairpin 1 show the narrowest conformational space (Fig. 7E and F). As previously described *via in vitro* methylation assays, METTL16 requires the structured hairpin to catalyze the methyl transfer.^12^ This is possibly mediated by a narrowing of the possible conformations of the substrate, which favors a correct position of the ADE:N6 in the catalytic pocket in a productive conformation.

**Fig. 10:**
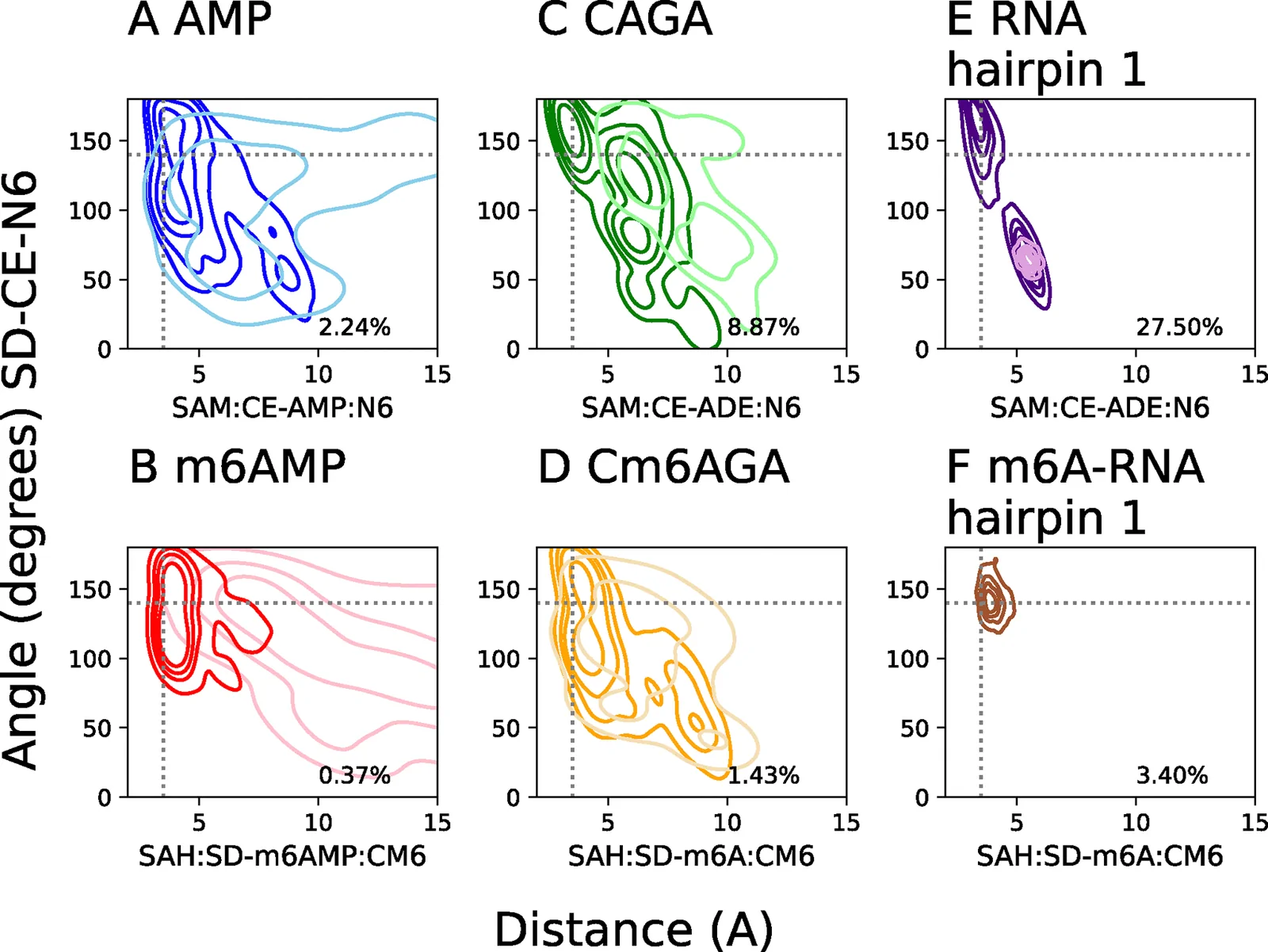
SAM-Ade conformational space narrows with increasing RNA length. Monomeric substrate and product show a wide range of conformations in the SAM-AMP (A) and SAH-m6AMP (B) distance and angle, both in the unbound and bound states. Increasing product/substrate length as for the tetranucleotide CAGA RNA (C) and Cm6AGA RNA (D) shows less variability. Short distance and restricted angle range are observed for the RNA hairpin 1 (E), and m6A-RNA hairpin 1 (F). Darker color indicates bound substrate or product, *i.e.*, a distance P185:O–A:N6 < 5 Å. The percentage of frames with a distance <3.5 Å and an angle >140° is shown in the lower right.

## 3. Methods

### 3.1. Chemical synthesis of bisubstrate analogs (BAs)

The synthesis of the BAs was as previously described by Atdjian *et al.*^21^

### 3.2. METTL16 production

Hexahistidine-tagged METTL16(1–291) was cloned into a pET-duet plasmid vector using the BamHI and XhoI restriction enzymes. The protein was overexpressed in Rosetta (DE3) *E. coli* cells over night at 18 °C, induced with 0.5 mM Isopropyl β-d-1-thiogalactopyranoside (IPTG) at OD600 above 1.0. The cells were harvested by centrifugation at 4000*g* for 15 minutes and the pellets stored at −80 °C until purification.

For the purification of METTL16(1–291), the frozen cell pellets were thawed on ice and resuspended in lysis buffer (50 mM HEPES pH 7.5, 500 mM NaCl, 5% Glycerol, 0.5% Tween-20, 1 mM DTT, Protease inhibitor cocktail (Roche) and salt-active GENIUS nuclease (ACROBiosystems)). The cells were lysed using a Maximator High Pressure Homogenizer at 40 kpsi. The lysate was clarified by centrifugation at 48 000*g* for 1 h and filtered through 0.45 µm filters before loading on a HisTrap HP nickel affinity column (Cytiva) equilibrated with equilibration buffer (50 mM HEPES pH 7.5, 500 mM NaCl, 5% Glycerol, 0.5% Tween, 1 mM DTT). The column was washed with 20 column volumes (CV) wash buffer containing 20 mM Imidazole. The protein was eluted with 400 mM Imidazole. The hexahistidine tag was cleaved by 0.1 mg mL^−1^ TEV protease over-night at 4 °C while dialyzing the protein against dialysis buffer (50 mM HEPES pH 7.5, 500 mM NaCl, 1 mM DTT) to reduce the imidazole concentration. The dialyzed protein solution was reapplied to the HisTrap HP column to separate the cleaved protein from the histidine-tagged TEV protease and the cleaved hexahistidine tag. The protein was further purified by size-exclusion chromatography on a HiLoad 16/600 Superdex 75 pg gel filtration column (Cytiva) in size-exclusion buffer (25 mM HEPES pH 7.5, 100 mM KCl, 50 mM NaCl, 1 mM TCEP). The purified protein was concentrated to 27 mg mL^−1^ using Amicon Ultra Centrifugal Filters with 10 kDa cutoff and subsequently filtered through 0.22 µm syringe filters.

### 3.3. Protein crystallization

METTL16(1–291) crystals were obtained in similar conditions as previously reported.^17^ 4 µL protein at 27 mg mL^−1^ was mixed with 2 µL reservoir solution (1.435 M K_2_HPO_4_, 45 mM NaH_2_PO_4_) and incubated in a hanging drop set-up at 21 °C. Crystals grew after 3 days. To soak the crystals with BA2 and BA4 the crystals were transferred into a drop containing 1.435 M K_2_HPO_4_, 45 mM NaH_2_PO_4_, 25% ethylene glycol and 25 mM BA2 or BA4 and incubated for 3 h. The crystals were then harvested and flash-frozen in liquid nitrogen.

### 3.4. Data collection and structure solution

Diffraction data was collected at the P13 beamline of the Deutsches Elektronen-Synchotron (DESY) in Hamburg, Germany. The data were processed using the autoPROC software package.^41^ The structures were solved by molecular replacement with phaser, using the apo-crystal structure of METTL16(1–291) (PDB: 6B91) as search model. Model building and structure refinement were perfomed using COOT^42^ and phenix.refine.^43^ Data collection and refinement statistics are shown in Table S1.

### 3.5. MD simulations

The crystal structure of the methyltransferase METTL16 in complex with BA4 was used as the starting conformation for the simulation of the pre- (SAM and AMP) and post-reaction (SAH and m6AMP) systems. The ligands were modified using the PyMOL^44^ editor, removing the linker atoms and mutating or rebuilding the missing ones. The regions missing from the crystal structure were “grafted” with the corresponding coordinates from the RNA-bound structure 6DU4 using PyMol. The grafted segments span M1-A9, P185-F227, and C276-D291. The BA4 ligand obtained from the crystal structure was then edited, to obtain either the pre- or post-reaction states. The AMP and m6AMP both contain a phosphate group. The obtained structures were then relaxed using the CAMPARI^45^ software to prepare them for simulation with GROMACS 2021.5.^46^ The CHARMM36m force field^47^ was used with the modified TIP3P model of water. CHARMM36m, despite some limitations in the description of nucleic acid interactions,^48^ has been shown to be a valuable force field for studying protein–RNA interactions, allowing the RNA to sample varied conformations.^49^ CHARMM36m does not have defined m6A parameters, therefore we have employed a CAMPARI-based tool^50^ to prepare the complex with the product and generate the GROMACS input files. This tool uses CGenFF v5.0^51^ to create the parameters for the m6A. For the substrate/METTL16 complex, the input files were generated manually using the pdb2gmx command, solvated into a 8.27 nm square box. Na^+^ and Cl^−^ ions were added up to 150 mM concentration, and an energy minimization was performed. The automatically prepared product/METTL16 complex was solvated into a 8.55 nm cubic box, with ions added as in the manual preparation, and subjected to a CAMPARI minimization, followed by a standard GROMACS energy minimization. From this point, both systems were handled in the same way.

For each simulation system, four runs were carried out independently. First, a 1 ns NPT equilibration was used to reach a 300 K and 1 bar temperature and pressure using the velocity^52^ and stochastic cell rescaling^53^ algorithms. Afterwards, a stepwise canonical equilibration with decaying restraints was applied to reduce clashes. The equilibration consisted of five equilibration steps of 3 ns each, with force constants halving from 10 to 0.67 kcal mol^−1^ Å^−2^ on the Cα atoms of residues 6–186 and 223–275, which were the ones resolved in the BA4 crystal structure. A final 5 ns step was applied with force constants of 0.38 kcal mol^−1^ Å^−2^ on the previously defined CA but also in the N6 and SD atoms of the AMP and SAM respectively. These restraints therefore allow the grafted sections of the protein to relax, while keeping the crystal structure stable. The decaying force constants allow the whole protein to relax slowly.

After this, an initial set of four production runs without any restraints were sampled for 100 ns. The displacement of the small molecules were monitored visually and with the use of RMSD, and the most stable copy was selected as a seed for longer production runs. Using as starting point the end of the equilibration of the most stable copy, 12 new production runs were conducted for 500 ns. METTL16 in the apo state was also simulated and treated in the same way as the substrate/METTL16 complex. For the apo state, since we started from a holo crystal structure, we first ran 200 ns to relax the effects of the ligands in the binding pocket, after which an additional 500 ns were sampled.

Finally, to study the effects of relevant single-point mutations on the dynamics of the substrates/products, the simulations were repeated using the same protocol for the respective system using the following mutants: F187A (SAM/AMP) and R282A (SAM/AMP). The systems sampled and the number and length of simulations obtained for each are summarized in Table 2.

**Table 2:** Summary of MD simulations performed

System name	Ligands	Sampling (ns)	Initial structure
RNA hairpin 1	SAM, MAT2A hairpin 1	2 × 500	2× independent equilibrations
m6A-RNA hairpin 1	SAH, MAT2A (m6A17)hairpin 1	2 × 500	2× independent equilibrations
CAGA	SAM, 5′-CAGA-3′	16 × 500	Equilibration #1–3
Cm6AGA	SAH, 5′-Cm6AGA-3′	16 × 500	Equilibration #1–4
AMP	SAM, AMP	4 × 100	4× independent equilibrations
		18 × 500	Equilibration #2
m6AMP	SAH, m6AMP	4 × 100	4× independent equilibrations
		18 × 500	Equilibration #2
apo	—	12 × 500	12× independent equilibrations
CAGA no SAM	5′-CAGA-3′	16 × 500	Equilibration #1–4
F187A	SAM, AMP	4 × 100	4× independent equilibrations
		6 × 500	Equilibration #3
R282A	SAM, AMP	4 × 100	4× independent equilibrations
		6 × 500	Equilibration #2 and #3

Given that METTL16 binds only structured RNA,^12^ we also simulated METTL16 in company of a longer RNA segment, 5′-CAGA-3′. We used the structure available with the MAT2A hairpin 1 (PDB code: 6DU4) as basis. We simulated the pre-catalytic state (SAM-CAGA), post-catalytic (SAH-Cm6AGA), and CAGA without SAM. The preparation of the simulations was analogous to the monomeric nucleotides. The experimentally determined structure of the hairpin was truncated to the relevant tetranucleotide. To further evaluate R-loop dynamics upon binding, MD simulations of the complex of METTL16 with the hairpin-RNA (PDB ID: 6DU4) were conducted in both the reactant and product states.

For the analysis, we were interested in calculating the unbinding times from METTL16. Therefore, we performed a fitting of the unbinding times of AMP, m6A, SAM, and SAH. The unbinding of the adenine was defined as the time of simulation when the distance between P185:O–A:N6:N6 became larger than 7.5 Å. The unbinding of SAM/SAH is described by the mean of the following distances: T164:O–SAM:N6, R82:CZ–SAM:C, N184:CG–SAM:C, E133:OE1–SAM:O3′, and E133:OE2–SAM:O2′. It is considered unbound when the value of the mean exceeds 7.5 Å. The frequency of bound molecules was calculated for each time point. The fitting was done using both a scaled exponential decay *a* × e^−*t*/*τ*^ and a two-phase model *a* × e^−*t*/*τ*_slow^ + (1 − *a*) × e^−*t*/*τ*_fast^. We report the mean lifetime *τ* obtained from the scaled relaxation time constant (Table 1).

All distance calculations were done using the mdtraj package. Root mean-squared fluctuations were calculated using a 10 ns sliding window to obtain average atom positions.

### 3.6. QM calculations

QM/MM free energy simulations were performed to probe the mechanism of METTL16 catalyzed methyl transfer reaction between SAM and N6 position of the adenosine substrate from both AMP and RNA. The N6 position is kept protonated in this study as previous QM/MM calculations suggested that the deprotonation of N6 occurred after the methyl transfer.^25^ The QM region included the cofactor SAM, the adenosine substrate, E133, N184, P185, the carbonyl group of T132 and P186. Link hydrogen atoms were added between C and Cα of T132, E133, C183 and P186 to saturate the valence of the QM boundary atoms. Additional link hydrogen atoms were added between C4 and C5 of ADE17 and GUA18 in SAM-RNA hairpin 1 simulations. The QM atoms were treated with the GFN2-xTB method.^38^ Benchmark calculations using model compounds indicate that the GFN2-xTB method describes the energetics of the methyl transfer reaction in good agreement with DFT calculations with a large basis set (see SI). The MM region was described with the CHARMM36m force field for proteins.^47^ The QM/MM simulations were carried out using the AMBER program^54^ with the xTB program patched^55^ to perform QM calculations.

Initial conformations of METTL16 complex with AMP and RNA were derived from the BA2 crystal structure and the RNA-bound structure (PDB ID: 6DU4), respectively. Energy minimization was performed on both systems using the CHARMM36m force field with GROMACS. Following energy minimization, both systems underwent a 12 ns stepwise equilibration in the canonical (NVT) ensemble. The equilibration process consisted of six consecutive 2 ns simulations with harmonic position restraints applied to the protein and RNA backbone atoms, the SD atom of SAM, and the N6 atom of AMP. The corresponding force constants were systematically reduced from 50 to 20, 10, 5, 2, and 1 kJ mol^−1^ Å^−2^. The resulting structure was used as the starting conformation of QM/MM simulations. Minimization of the QM region was carried out with the MM region under harmonic position restraint, followed by a 100 ps equilibration to further relax the QM region. During the equilibration, the temperature was ramped up from 100 K to 300 K in the first 50 ps and kept at 300 K in the remaining 50 ps using Langevin thermostat.^56^The real space cutoff of electrostatic interaction was set to 1.2 nm. Long-range electrostatic interactions were treated using the particle-mesh Ewald method.^57^ SHAKE algorithm was applied to all bonds involving hydrogen and used to avoid undesired proton transfer reactions.^58^ An integration timestep of 2 fs was used in all QM/MM simulations.

To probe the energetics of the methyl transfer reaction catalyzed by METTL16, well-tempered multiple-walker metadynamics simulations^59,60^ were carried out using PLUMED.^61^ The antisymmetric stretch, which describes the difference between the C–S distance and C–N distance from adenosine N6 position and SAM sulphur methyl group was chosen as the collective variable (CV), *i.e.*, *ξ* = *r*(CE–SD) − *r*(CE–N6). The individual distances and S–C–N bond angle were also recorded but no bias potential was added. An upper/lower wall with a force constant of 10 kJ mol^−1^ Å^−2^ was placed at *ξ* = 1.7 Å/*ξ* = −1.7 Å to help sample conformations only relevant to methyl transfer reaction. Prior to the production runs, a non-tempered metadynamics run was carried out to roughly estimate the barrier height and efficiently sample conformations along the transition path. Conformations located near the reactant and product states were chosen as the initial states of the subsequent multiple-walker simulations. In the production well-tempered runs, Gaussian bias potential was deposited at the frequency of 0.2 ps with an initial height of 1.0 kJ mol^−1^, width of 0.05 Å and a bias factor of 35. Ten walkers with different initial states (both positions and velocities) chosen from the non-tempered run, as described previously, were used in production while sharing bias deposited among all walkers every 1 ps. Each walker was run for 300 ps for a total of 3 ns of sampling to obtain the potential of mean force (PMF). Convergence was evaluated by comparing the PMF profile as a function of the simulation time (see SI).

## 4. Conclusions

We have studied the (co)substrate recognition and catalytic mechanism of METTL16 by a combination of experimental techniques, classical MD and QM/MM methods. We first solved the crystal structures of the MTD domain of METTL16 in complex with two BAs (at resolution of 2.2 Å and 2.3 Å, respectively). The comparison of the BA/METTL16 crystal structures with METTL16 complexes with SAH or RNA-hairpin demonstrates that the binding mode of the BA mimics the transition state of methyl transfer. We then carried out MD simulations started from apo METTL16 and complexes with oligonucleotide-RNA modeled on the BA/METTL16 structures. We observed unbinding of SAM on the 1-µs time scale which is in agreement with the *K*_m_ value in the high micromolar range. These simulations showed substantial displacements of the N-terminal segment and the R-loop (residues 189–213). The large flexibility of the R-loop, which includes three arginine side chains (Arg200, Arg203, and Arg204), is likely to capture the RNA-substrate by a fly-casting mechanism and long-range electrostatic interactions. We propose the fly-casting mechanism as a general means of recruiting charged biopolymers by protein loops with side chains of opposite charge. Furthermore, the MD simulations reveal that the motion of the side chain of F187 is coupled to the binding of the substrate adenine. The presence of the substrate hairpin 1 RNA stabilizes SAM in its recognition pocket, and significantly rigidifies the R-loop and N-terminal segment of METTL16.

QM/MM free energy calculations show that the methyl transfer reaction does not require prior deprotonation of adenosine-N6, similar to the main m6A-RNA writer enzyme METTL3–14. Despite this similarity, electrostatic interactions between the charged groups of SAM and charged side chains in the catalytic pocket are more prominent for METTL3–14 than METTL16; substrate stabilization by nearby non-polar and aromatic residues also differs between the two active sites. As a consequence, the free energy profile of the methyl transfer reaction shows a significantly lower barrier for METTL3–14 than METTL16. However, the height of the barrier of the reaction catalyzed by METTL16 indicates that the rate-limiting step is not the catalytic step, but rather the dissociation of SAH and/or the RNA product. METTL3–14 has a relatively high affinity for SAM and does not require the RNA substrate to be structured.^62^ Thereby, it is optimized for efficient methylation of any free adenosine within the consensus GGACU motif of a large number of mRNA substrates.^63^ By contrast, METTL16 has a weak affinity for SAM and depends on a structured RNA substrate for methyl transfer. These properties limit the catalytic efficiency of METTL16 but enable methylation under conditions that reflect cellular SAM levels, thereby coupling catalysis to metabolic sensing.

Our combined experimental and simulation study sheds light on the intrinsic plasticity of METTL16, the rigidification upon RNA-substrate binding, and the catalytic mechanism. The crystallography data on bisubstrate analog binding, the influence of RNA-substrate size on the flexibility of METTL16 as revealed by MD, and the QM/MM analysis of the methyl transfer reaction are useful for the design of small-molecule inhibitors.

## Author contributions

F. S., Z. W., P. V.-R.: investigation, visualization, writing – original draft, writing – review & editing; I. C.: validation, writing – review & editing; E. B., L. I.: investigation, resources; Q. C., A. C.: conceptualization, supervision, funding acquisition, writing – review & editing.

## Conflicts of interest

There are no conflicts to declare.

## Supplementary Material

CB-OLF-D6CB00242K-s001

## Data Availability

Crystallographic data for METTL16 in complex with BA2 and BA4 have been deposited in the Protein Data Bank under accession codes 28WE (https://www.rcsb.org/structure/28WE) and 28ZC (https://www.rcsb.org/structure/28ZC) respectively. Coordinates and topologies for MD simulations are available at Zenodo at repository 21489791 (https://doi.org/10.5281/zenodo.21489791). Supplementary information (SI): additional information on experimental and simulation procedures, including supplementary figures, are available in the document. See DOI: https://doi.org/10.1039/d6cb00242k.
