# Supplementary material for: Catalysis tuned for metabolic sensing: dynamics and mechanism of the RNA methyltransferase METTL16
Source: RSC Chem Biol. 2026 Oct 7. Online ahead of print. doi: 10.1039/d6cb00242k (PMC13690257; doi:10.1039/d6cb00242k)
Supplement: Supplementary file 1 [file CB-OLF-D6CB00242K-s001.pdf]

Supplementary Information (SI) for RSC Chemical Biology.
This journal is © The Royal Society of Chemistry

Catalysis Tuned for Metabolic Sensing: Dynamics and Mechanism of the RNA Methyltransferase METTL16

Supplementary Information

Fiona Stamm+,[†] Zhongyi Wan+,[‡] Pablo Vargas-Rosales+,[†] Ivan Corbeski,[†] Emmanuelle Braud,[¶] Laura Iannazzo,[¶] Qiang Cui,[*,‡] and Amedeo Caflisch[*,†]

†*Department of Biochemistry, University of Zurich, Winterthurerstrasse 190, 8057 Zürich, Switzerland*

‡*Department of Chemistry, Boston University, 590 Commonwealth Ave., Boston, MA, 02215, United States of America*

¶*Université Paris Cité, CNRS, Laboratoire de Chimie et Biochimie Pharmacologiques et Toxicologiques, Paris, France*

E-mail: qiangcui@bu.edu; caflisch@bioc.uzh.ch

1 Crystallography

Table S1: Crystallography data collection and refinement statistics. Statistics for the highest-resolution shell are shown in parentheses.

	**BA2 complex** (PDB 28WE)	**BA4 complex** (PDB 28ZC)
Wavelength (Å)	0.9762	0.9762
Resolution range (Å)	76.41 - 2.29 (2.33 - 2.29)	77.07 - 2.17 (2.21 - 2.17)
Space group	I 41 3 2	I 41 3 2
Unit cell (Å, °)	187.18 187.18 187.18 90.00 90.00 90.00	188.78 188.78 188.78 90.00 90.00 90.00
Total reflections	1876263 (46111)	2499484 (125317)
Unique reflections	25439 (1230)	30596 (1511)
Multiplicity	73.8 (37.5)	81.7 (82.9)
Completeness (%)	99.9 (99.7)	100.0 (100.0)
Mean I/sigma(I)	20.2 (1.2)	26.4 (1.0)
Wilson B-factor	59.42	59
R-merge	0.177 (5.303)	0.173 (12.718)
R-meas	0.178 (5.376)	0.174 (12.796)
R-pim	0.020 (0.861)	0.019 (1.401)
CC1/2	0.998 (0.442)	0.999 (0.374)
CC*	1 (0.78)	1 (0.74)
Reflections used in refinement	25390 (2691)	30587 (2747)
Reflections used for R-free	1261 (113)	1470 (135)
R-work	0.2037 (0.2877)	0.2075 (0.3215)
R-free	0.2229 (0.2961)	0.2339 (0.3626)
Number of non-hydrogen atoms	2138	2151
macromolecules	2033	2036
ligands	74	79
solvent	31	36
Protein residues	259	259
RMS bonds (Å)	0.007	0.008
RMS angles (°)	0.939	0.885
Ramachandran favoured (%)	97.24	97.25
Ramachandran allowed (%)	2.76	2.75
Ramachandran outliers (%)	0	0
Rotamer outliers (%)	0.98	0.99
Clashscore	5.6	3.89
Average B-factor	62.35	62.88
macromolecules	61.56	62.05
ligands	83.47	83.13
solvent	63.94	65.49

2 Classical Molecular Dynamics

2.1 Unbinding rate calculation

We used two forms of exponential decay to model the unbinding of the substrate/product and of SAM/SAH from their respective binding pockets. First, a two parameter model $a * e^{-t/\tau}$, which corresponds to a scaled exponential decay. The exponential mean lifetime τ is the value we report in Table 1 of the main text. A three-parameter model, of the form $(1-a) * e^{-t/\tau_{fast}} + a * e^{-t/\tau_{slow}}$, was used to corroborate the quality of the fitting. The parameters for the models are reported in Table S2 for the substrate and product unbinding events (flipping out of the adenine), and in Table S3 for the SAM/SAH.

Table S2: Parameters of the fittings for the exponential decay of the fraction of bound molecules at the substrate/product pocket in METTL16. The two parameter model is $a * e^{-t/\tau}$, and the three-parameter model is $(1-a) * e^{-t/\tau_{fast}} + a * e^{-t/\tau_{slow}}$.

System	Substrate/Product unbinding times (ns)				
	two-parameter model		three-parameter model		
	a	τ	a	τ_{slow}	τ_{fast}
CAGA / SAM	0.92	1162	0.75	3155	102
Cm6AGA / SAH	0.90	567	0.89	574	4
CAGA / -	0.95	338	0.93	347	15
AMP / SAM	0.88	388	0.85	409	14
m6AMP / SAH	0.63	90	0.57	99	2

Table S3: Parameters of the fittings for the exponential decay of the fraction of bound molecules at the SAM/SAH pocket in METTL16. Similar to Table S2

System	SAM/SAH unbinding times (ns)				
	two-parameter model		three-parameter model		
	a	τ	a	τ_{slow}	τ_{fast}
CAGA / SAM	1	970	0.48	>> 10000	401
Cm6AGA / SAH	1.04	2603	1.1	1884	90
AMP / SAM	1.04	774	1.05	757	15
m6AMP / SAH	1	>> 10000	2.05	>> 10000	>> 10000

Figure S1 shows the decay in bound fraction (solid lines) and the fitted two-parameter

exponential decay functions (dotted). For the substrate/product unbinding, most values are well modeled by the scaled exponential decay, except for the F187A mutant and the m6AMP (SAH) system. For the SAM/SAH unbinding, most pre-exponential scaling values are close to 1.

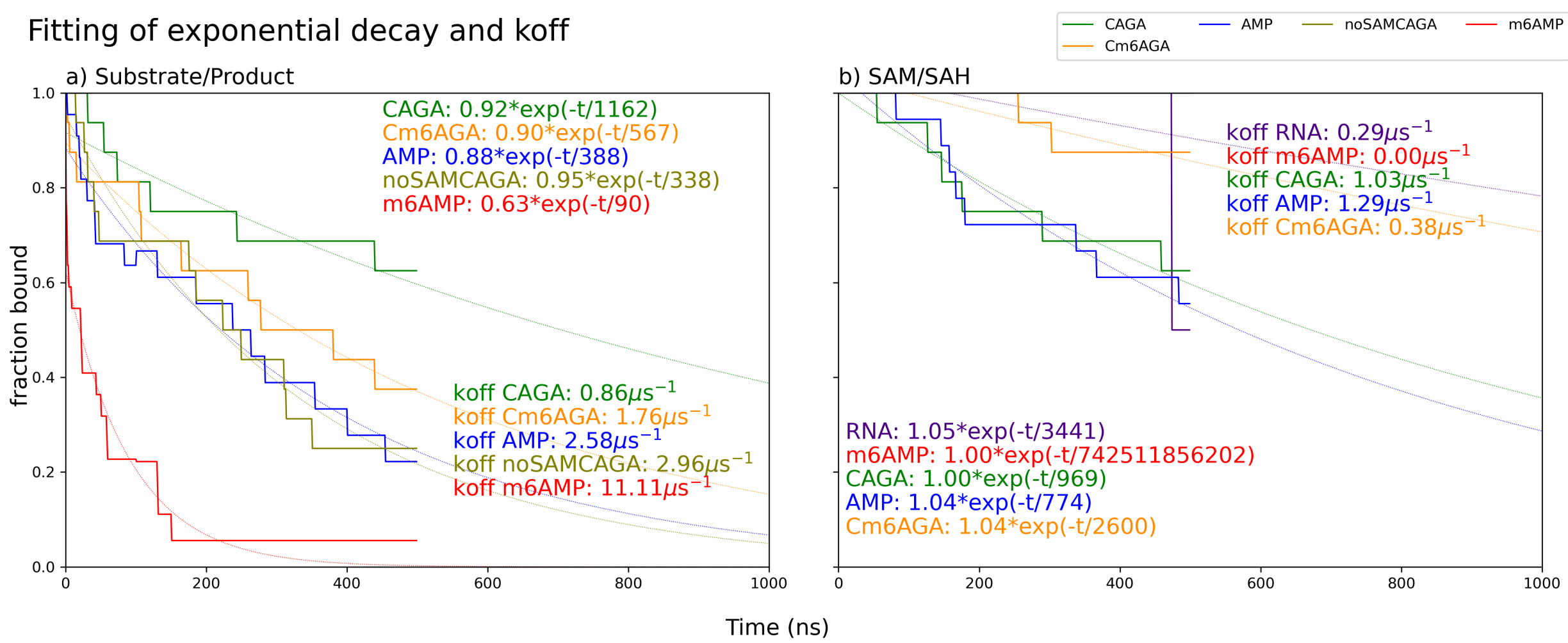

Figure S1: Fitting of the fraction (solid step-like trace) of bound susbtrate/product (a) or SAM/SAH (b) to scaled exponential decay functions (dotted lines). The fittings and the k_{off} values are shown in the legends.

The unbinding of SAM/SAH was monitored using a collective variable (CV) which consists of the mean of the following five distances between METTL16 and individual groups of SAM/SAH: T164:O-N6 (adenine ring), R82:CZ-C and N184:CG-C (methionine moiety), and E133:OE1-O3' and E133:OE2-O2' (ribose). The time series of the distances and the CV (mean) are shown here:

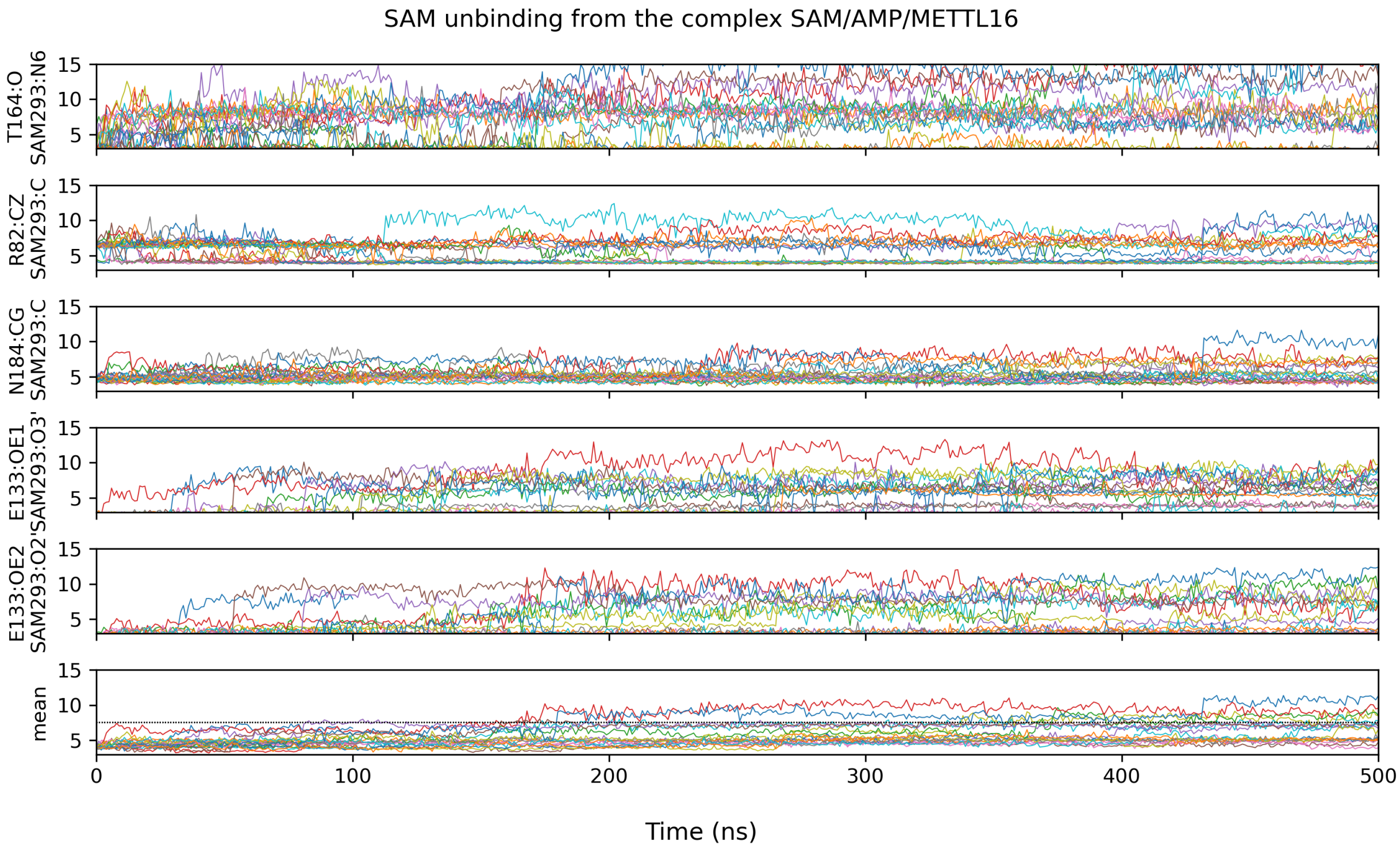

Figure S2: Monitoring of the unbinding of SAM in presence of AMP. Interatomic distances and CV (mean) used for the fitting of the exponential decay function. The horizontal line at 7.5 Å shows the unbinding threshold for the CV.

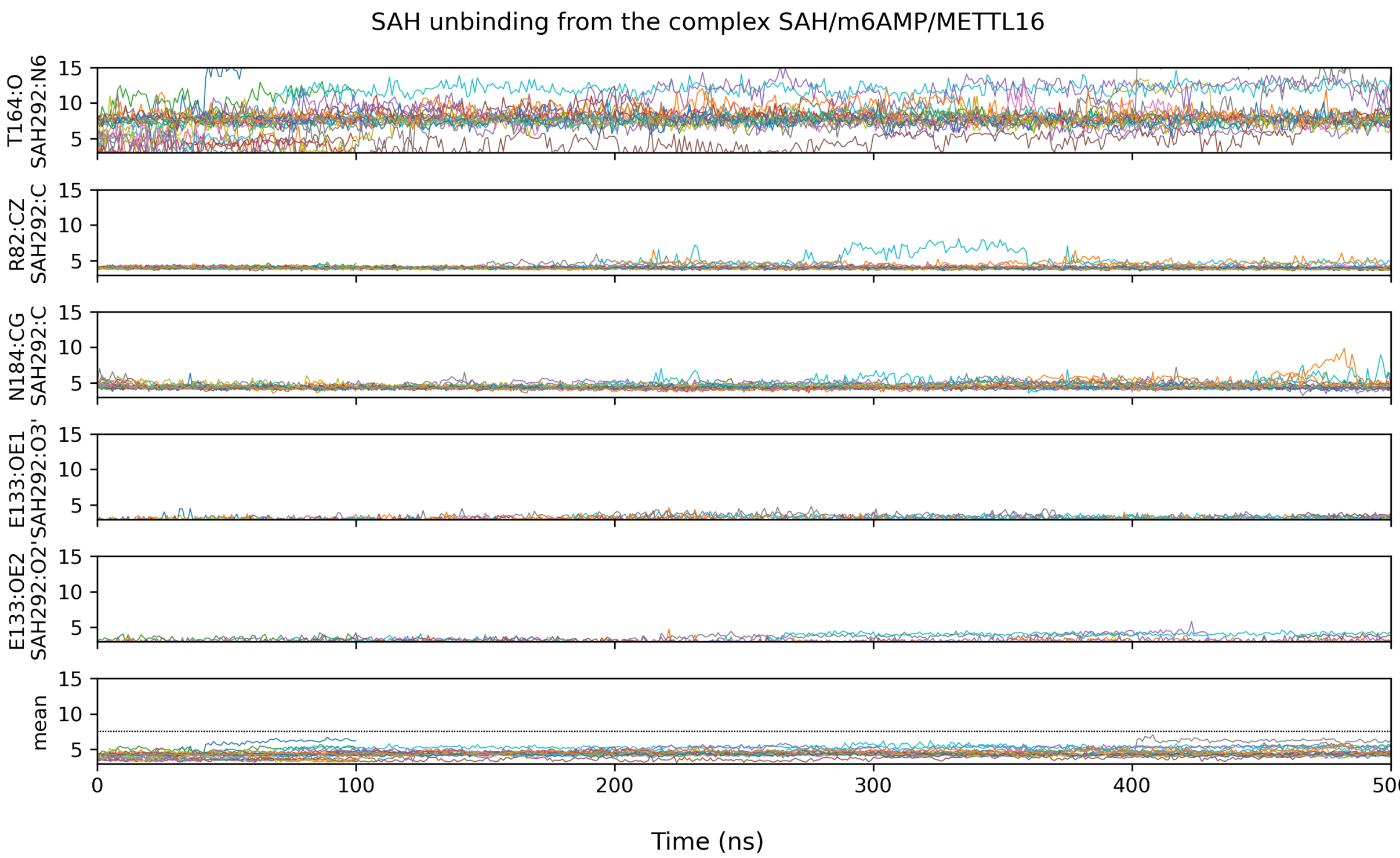

Figure S3: Same as Figure S2, regarding SAH unbinding in presence of m6AMP.

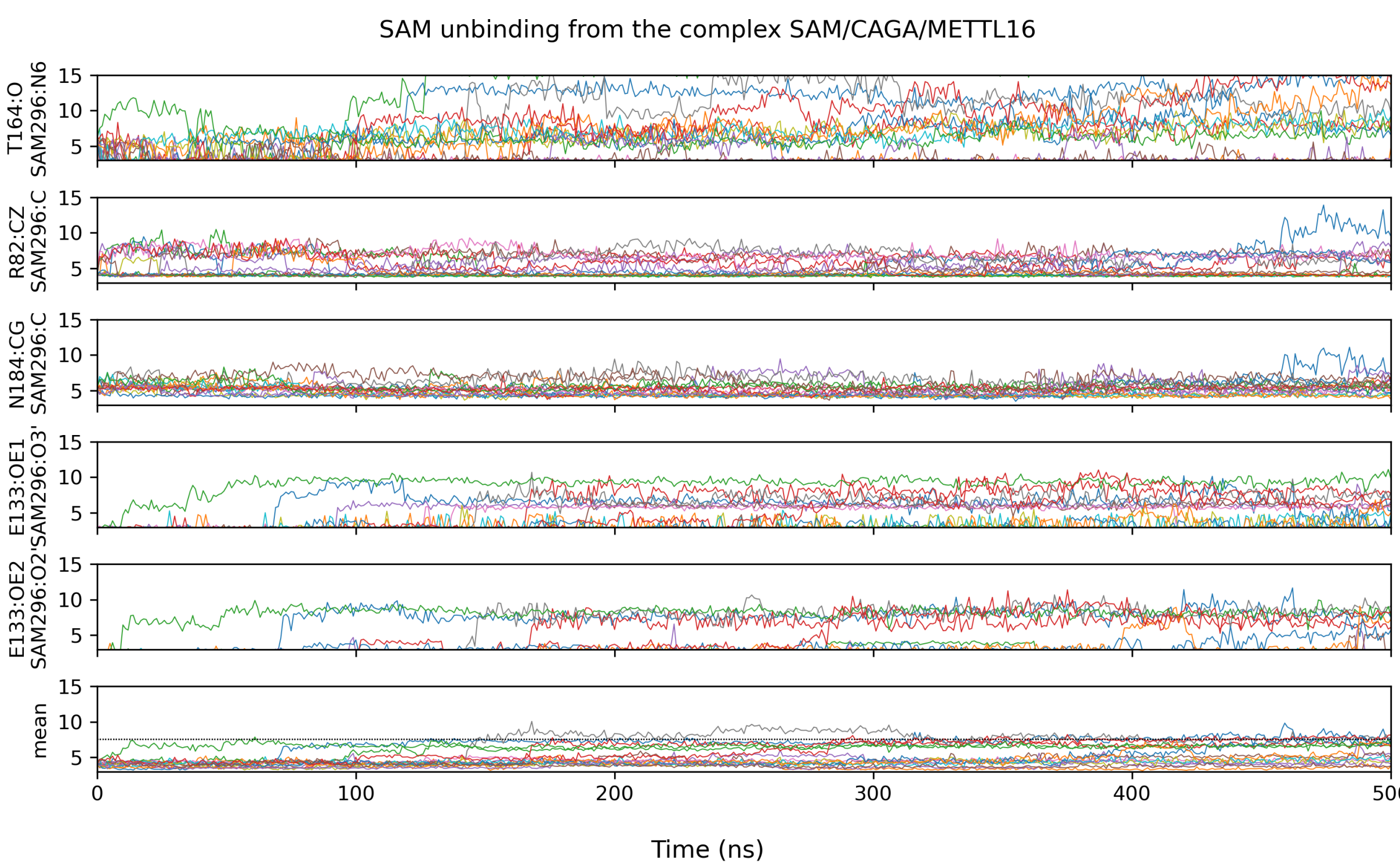

Figure S4: Same as Figure S2, regarding SAM unbinding in presence of CAGA.

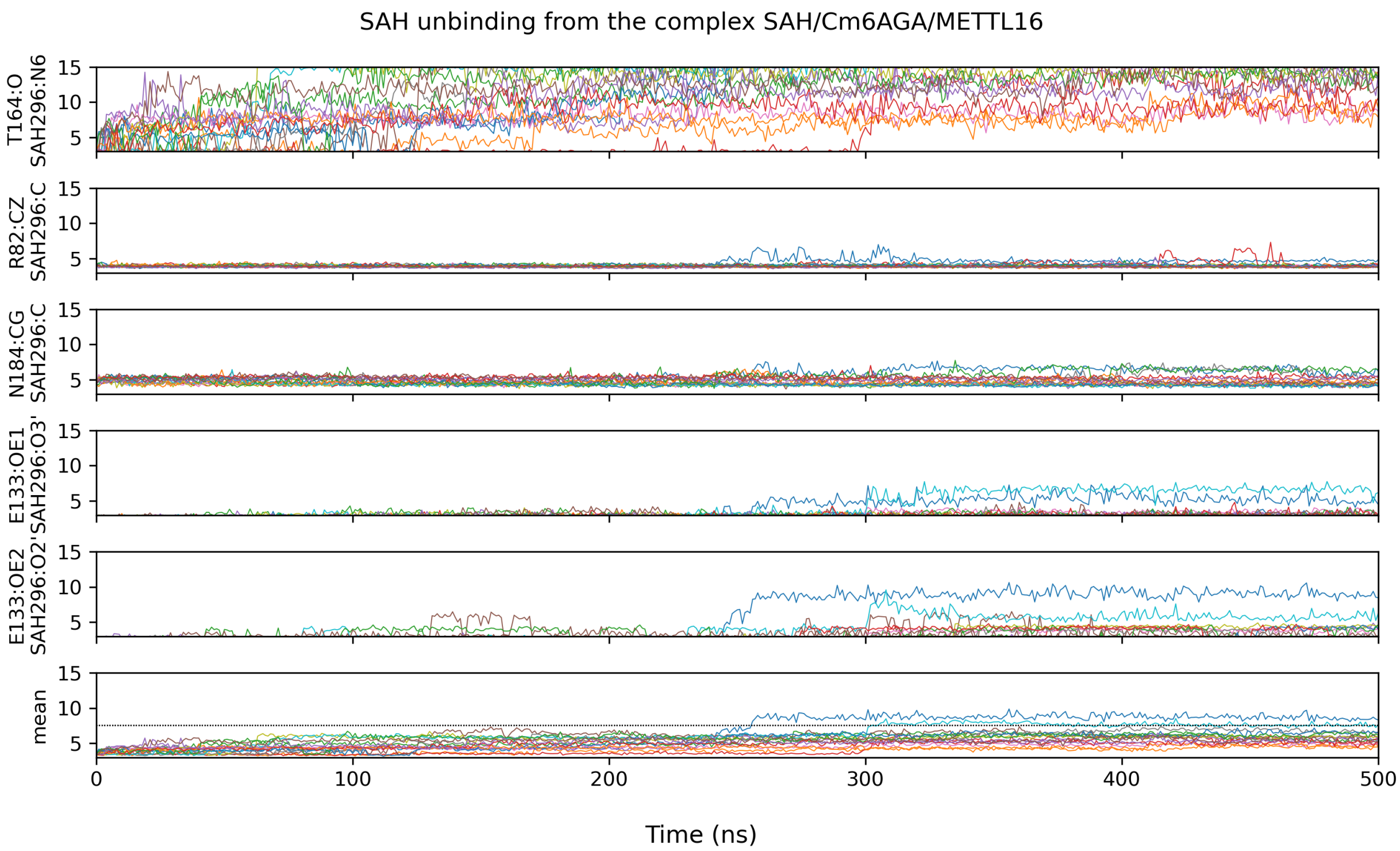

Figure S5: Same as Figure S2, regarding SAH unbinding in presence of Cm6AGA.

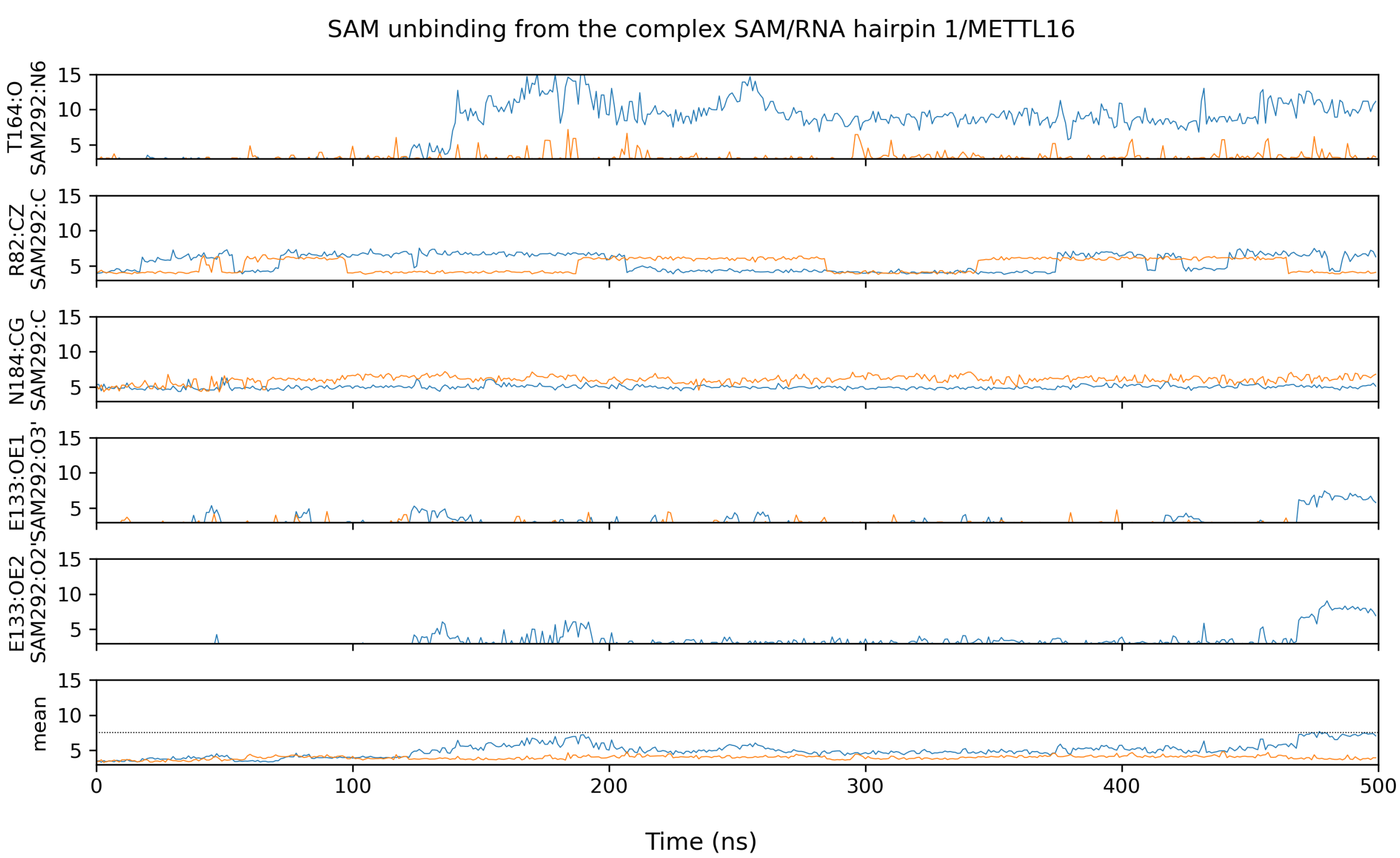

Figure S6: Same as Figure S2, regarding SAM unbinding in presence of RNA hairpin 1.

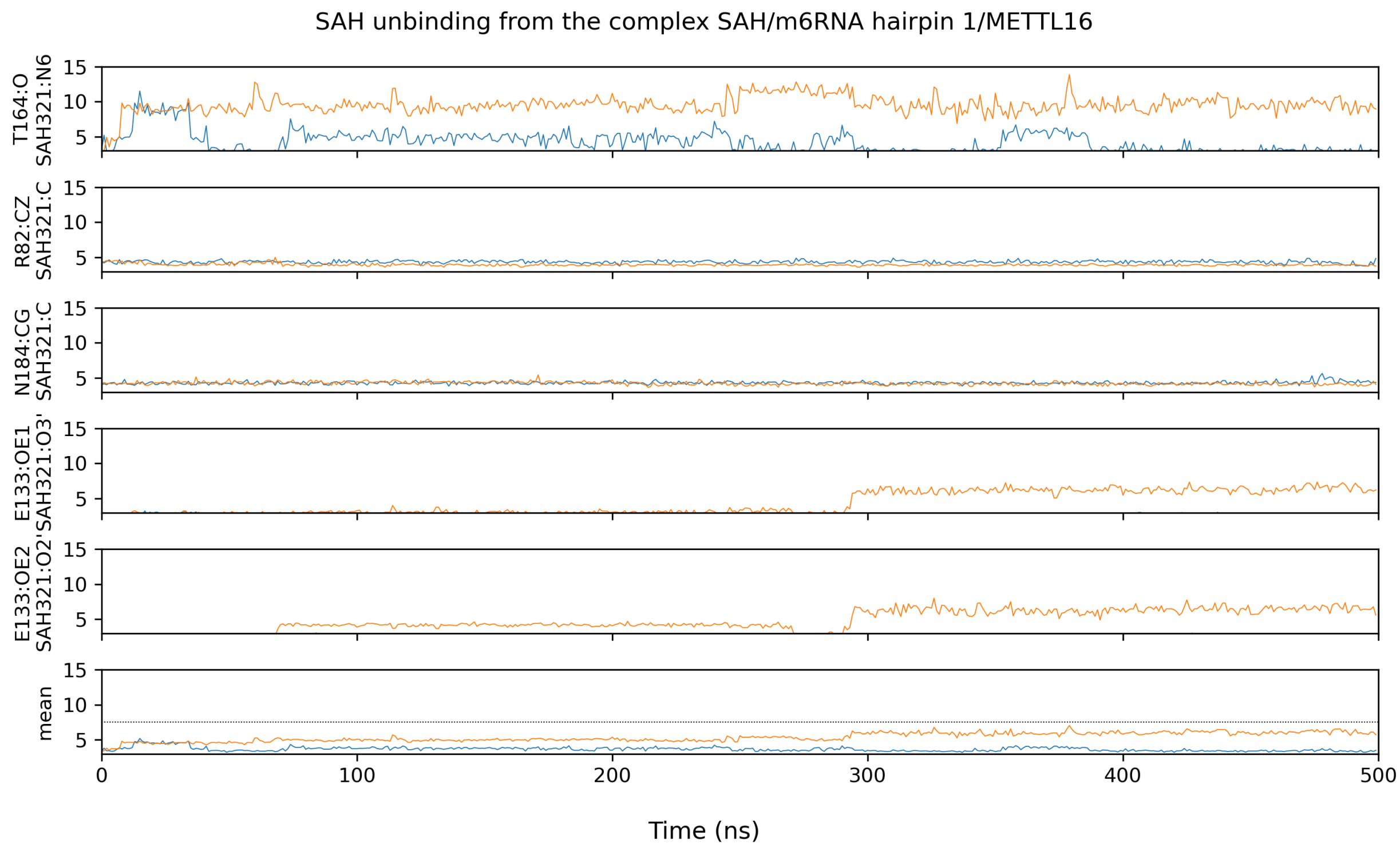

Figure S7: Same as Figure S2, regarding SAH unbinding in presence of m6RNA hairpin 1.

To allow for an individual analysis, we now show each of the trajectories containing unbinding events of the substrate/product (Figure S8). The substrate/product unbinding is defined as a distance between P185:O and N6 (blue trace) larger than 7.5 (red dotted line). We were interested in the coupling between F187 and the substrate/product. Figure 9 of the main text shows that the tetrameric RNA shows stronger coupling than the monomeric systems. We monitor the closeness of these two using the distance between the CZ atom of F187 and C5 of the substrate/product. The distance between F187:CZ and N184:CG shows the orientation of F187 in the pocket (green trace). It was theorized, that the F187 accompanies the substrate/product in its unbinding from the active site. We observe this behavior in trajectory 7 of CAGA, for example, where the exit of the adenine from the pocket (blue, increases) is accompanied by the concomitant flipping of F187 out of the pocket (green), while maintaining a close distance to the adenine (orange). A similar situation is seen in trajectory 5 and 12 of Cm6AGA, although in 5 the F187 eventually uncouples from

the N6-methyladenosine. In the monomeric AMP and m6AMP runs, there is no coupling observed, and indeed no π-π stacking can be seen, as in no case there is a stable interaction between F187 and the substrate/product.

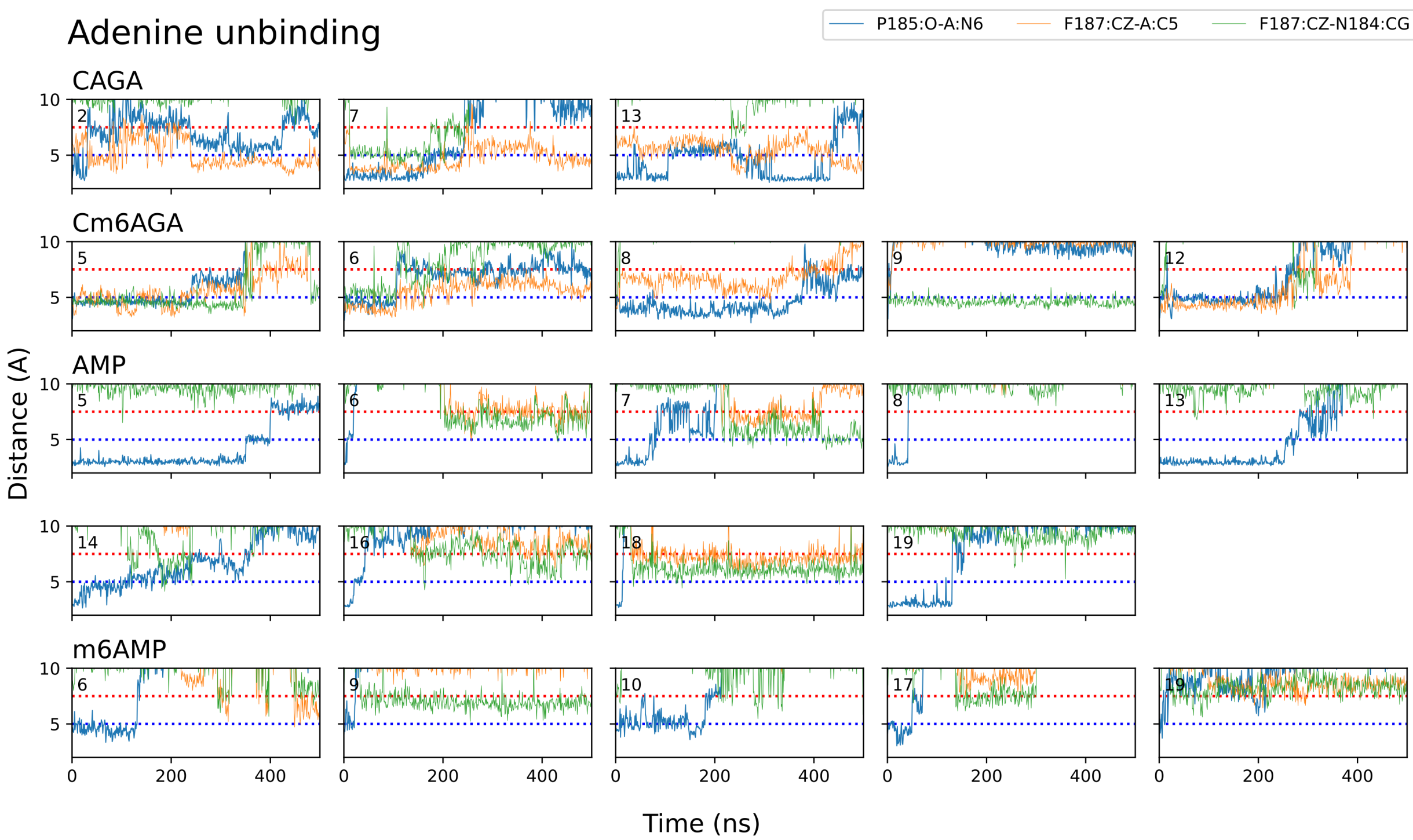

Figure S8: The unbinding of the substrate/product for the individual trajectories (number in the top left) is monitored by the P185:O-A:N6 distance (blue trace). The behavior of F187 is shown by its interaction with the substrate/product (orange) and its orientation in the pocket (green). The threshold at 7.5 Å(red dotted line) marks the unbinding. The threshold at 5 Åis considered rebinding (blue dotted line).

In the case of the substrate/product, some (partial) rebinding events were observed in the mono- and tetrameric runs. The specific unbinding events of the substrate/product are shown in Figure S9. Again, as was observed in the unbinding events, the π-π stacking between the substrate/product and F187 rings can be observed in the tetrameric systems only. Another interesting insight is that the F187 does not need to point towards the pocket to have a bound substrate/product.

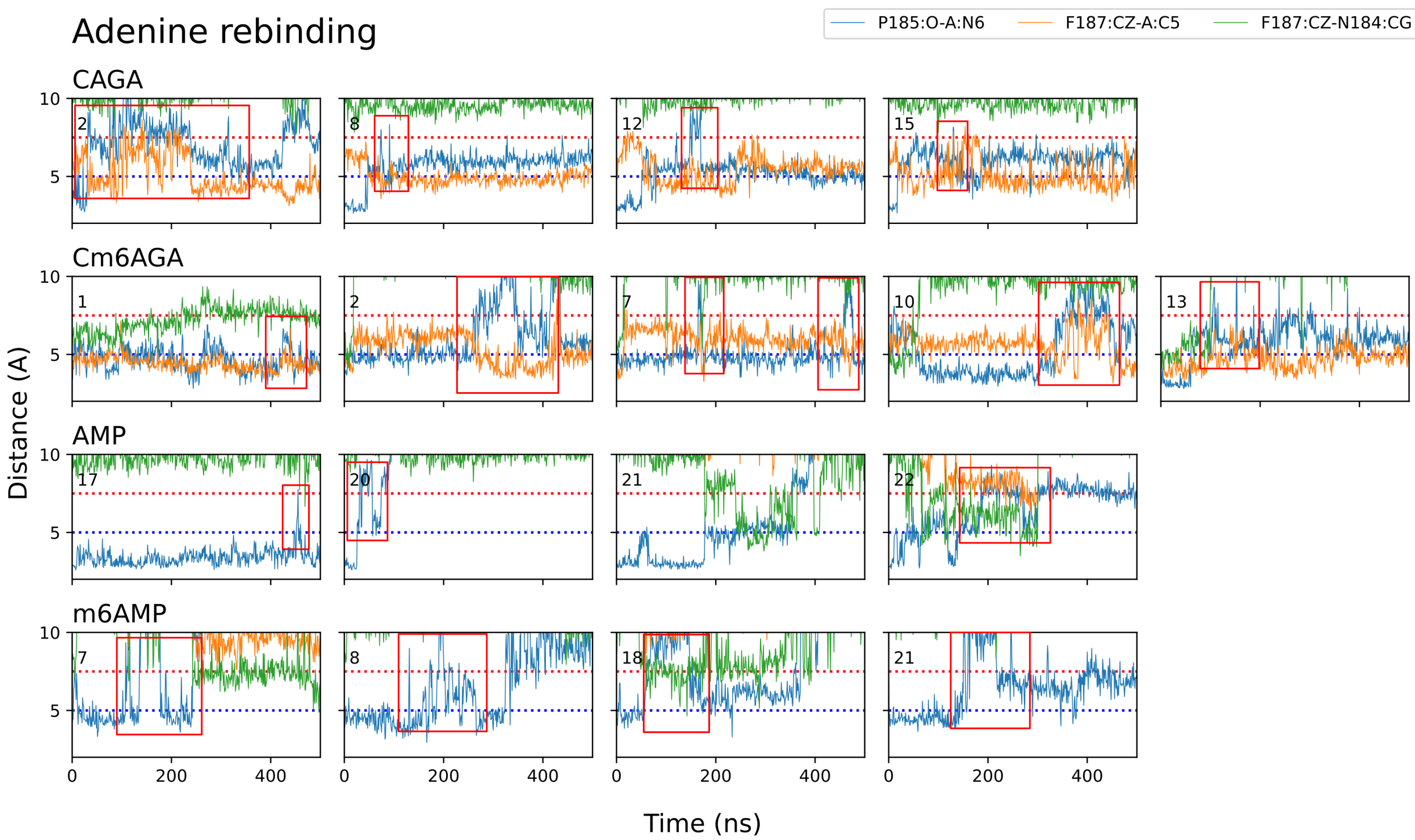

Figure S9: Same as Figure S8, but for rebinding events (red boxes).

2.2 Flexibility of METTL16

The Cα root mean squared fluctuation was calculated using an average conformation calculated for every 10 ns (sliding window). The exact calculation is described explicitly in the Supplementary Information of a previous publication.[1] As described in the experimental data, and as suggested by the QM/MM simulations, methylation by METTL16 needs not only the stabilization by the binding pocket residues but also by the RNA structure itself.

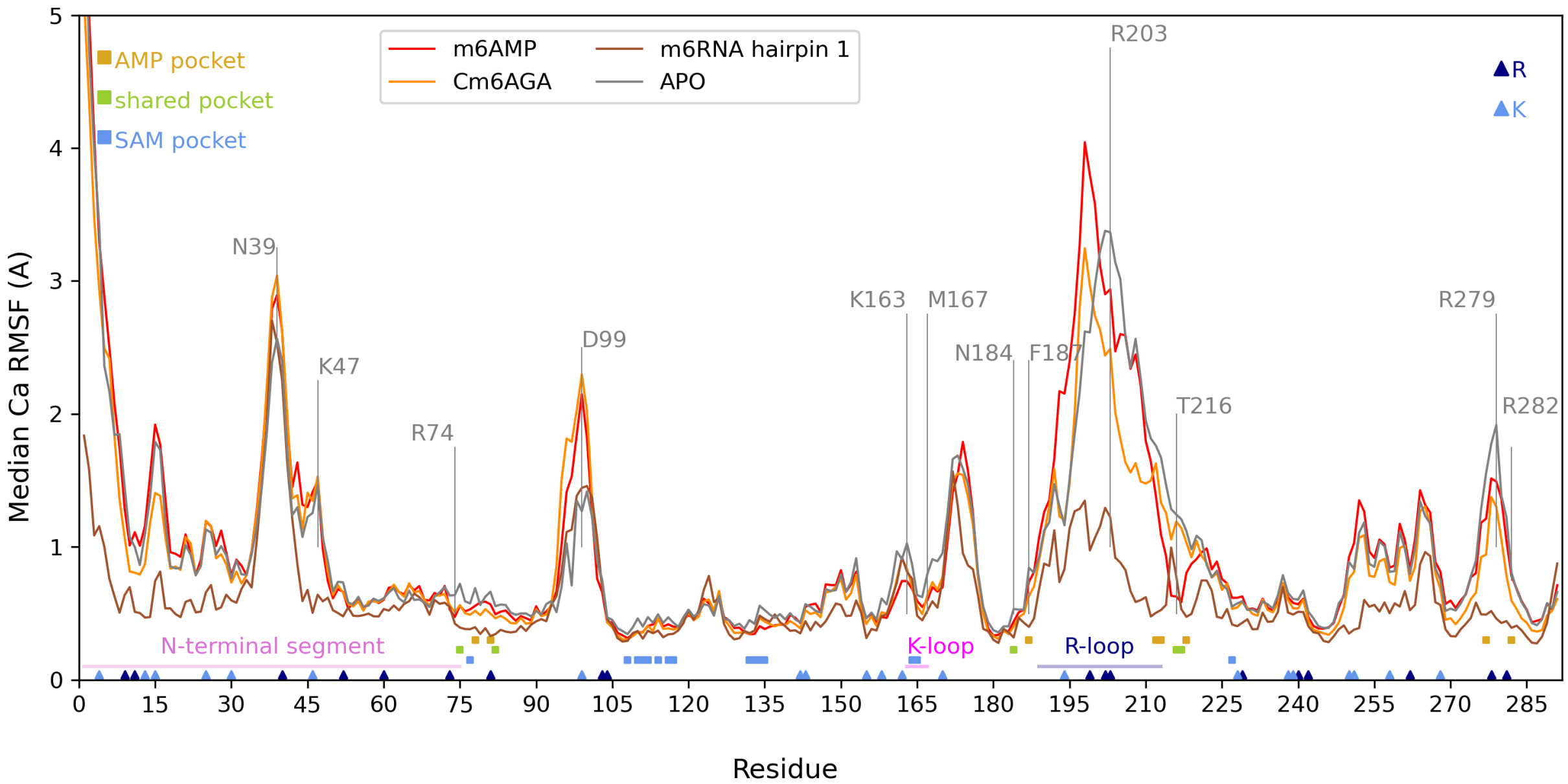

Figure S10: Median Cα root-mean squared fluctuations of METTL16 in the complex with product (m6AMP, Cm6AGA, m6ARNA) and apo. RMSF varies across systems, with higher stabilization observed in the complex with the hairpin RNA (m6ARNA $<$ m6AMP $\simeq$ CAGA $\simeq$ apo). Charged residues are marked as blue triangles (dark: R, light: K). Relevant structural regions of METTL16 are labeled by horizontal lines, and the binding pockets are marked by colored squares for the AMP, SAM, and shared pockets. Some interesting residues are explicitly labeled.

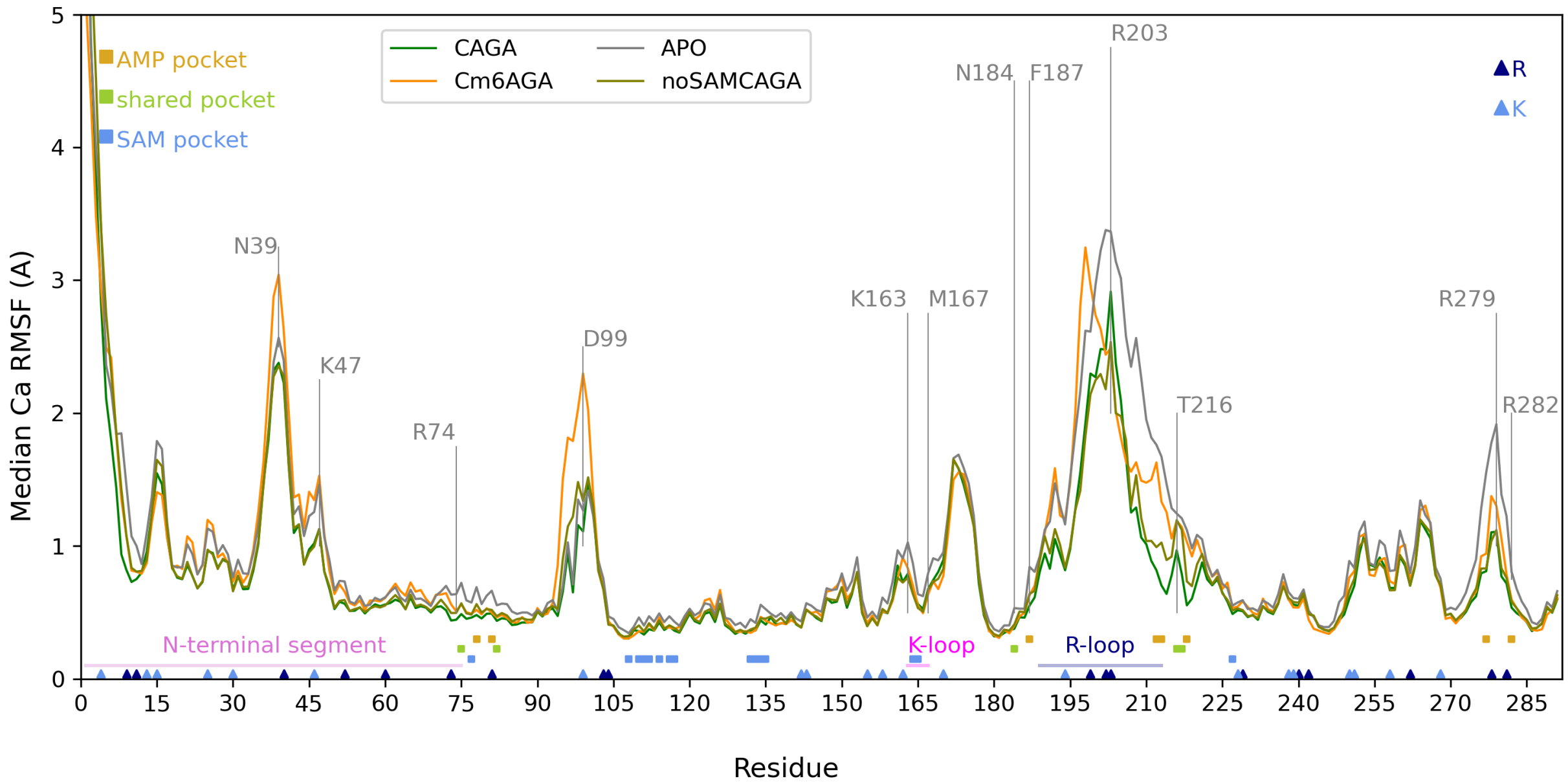

Figure S11: Median Cα root-mean squared fluctuations of METTL16 in the complex with tetranucleotide substrate/product. Labeling analogous to Figure S10.

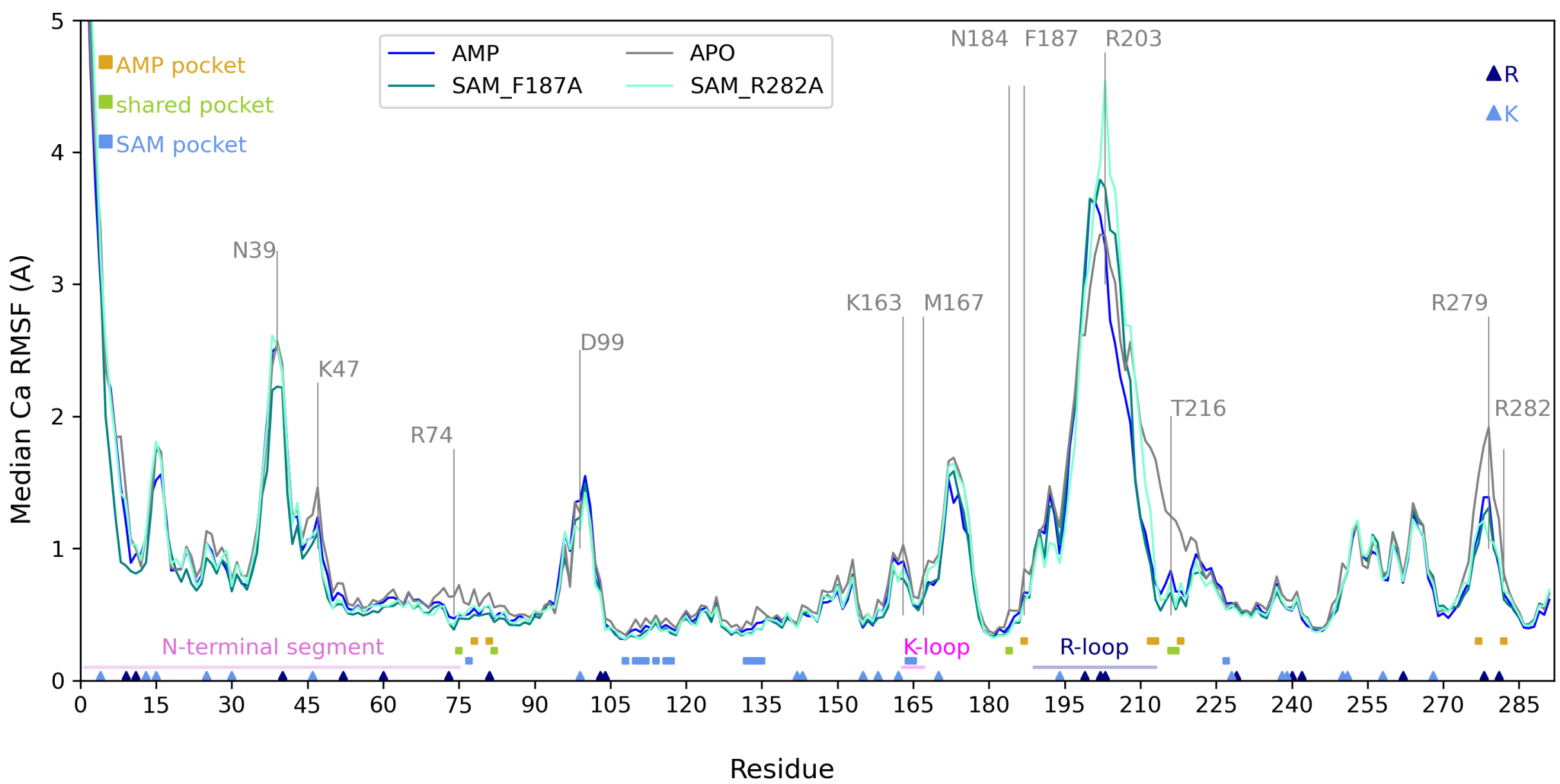

Figure S12: Median Cα root-mean squared fluctuations of METTL16 point mutants. Labeling analogous to Figure S10.

2.3 Analysis of individual distances: role of the K163 side chain and fly-casting mechanism of R-loop (residues 189-213)

Several distances were monitored throughout the simulations (Figure S13).

The distance K163:NZ-N184:O shows whether K163 blocks the SAM binding pocket. In no case, did we observe a distance comparable to the one in the hp1-bound crystal structure (6DU4) where K163 blocks the SAM binding pocket.

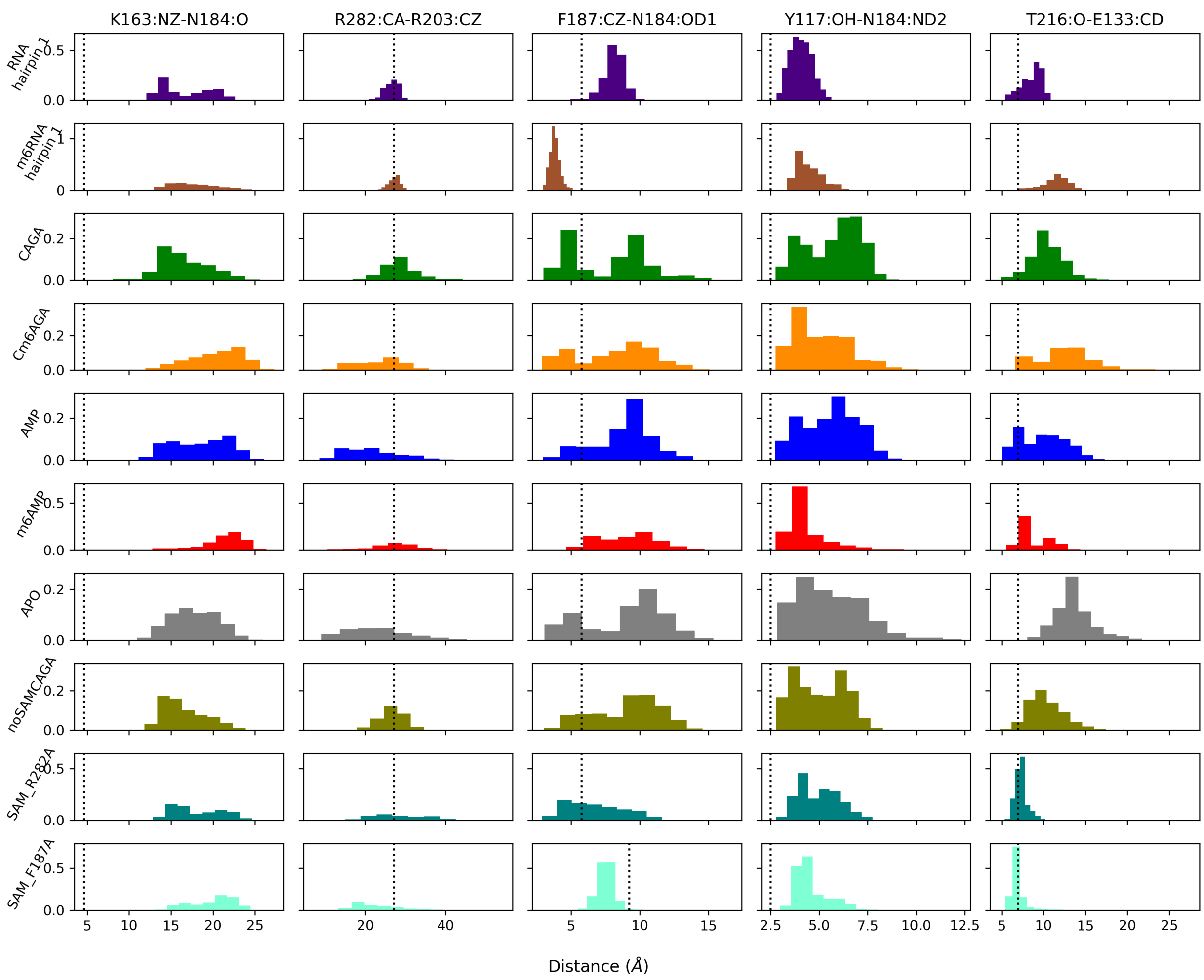

Figure S13: Histogram of interatomic distances. The selected distances in the crystal structure with MAT2A hairpin 1 (6DU4) are shown as dotted lines.

The fly-casting mechanism is supported by the high flexibility of the R-loop (residues 189-213). The electrostatic interactions between the positively charged Arg side chains in

the R-loop (R200, R203, and R204) recruit the RNA phosphates. In the simulations with the MAT2A hp1 (RNA and m6RNA hairpin 1) the distance is very constrained.

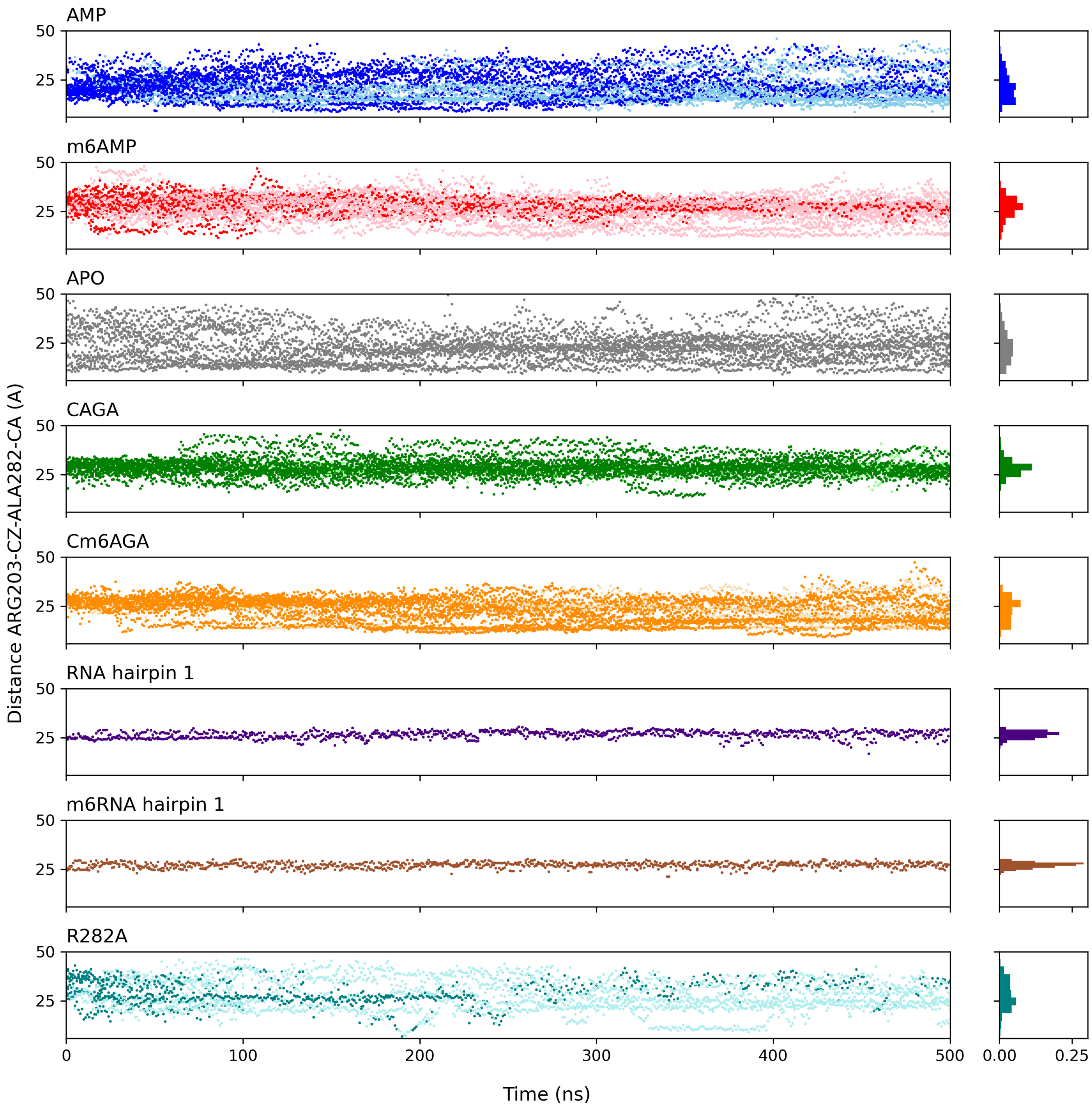

Figure S14: Time series of R203:CZ-R282:CA distances. Darker color indicates bound substrate/product.

The distance between F187:CZ-N184:OD1 (or A187:CB for the mutant) serves to measure the positioning of the F187 as companion for the substrate adenine through π-π stacking, as well as the correct orientation of N184, i.e. in a catalytically active conformation with

OD1 pointing towards the N6 of the substrate adenine. Related to this, as observed in the crystal structure with BA2, N184 can also form a hydrogen bond with Y117:OH, which is monitored by the distance to N184:ND2. This conformation, where N184:OD1 points towards the substrate and N184:ND2 points towards SAM, is the one that enables methyl transfer. A detailed structural analysis of N184 and its orientation can be seen in Figure S15.

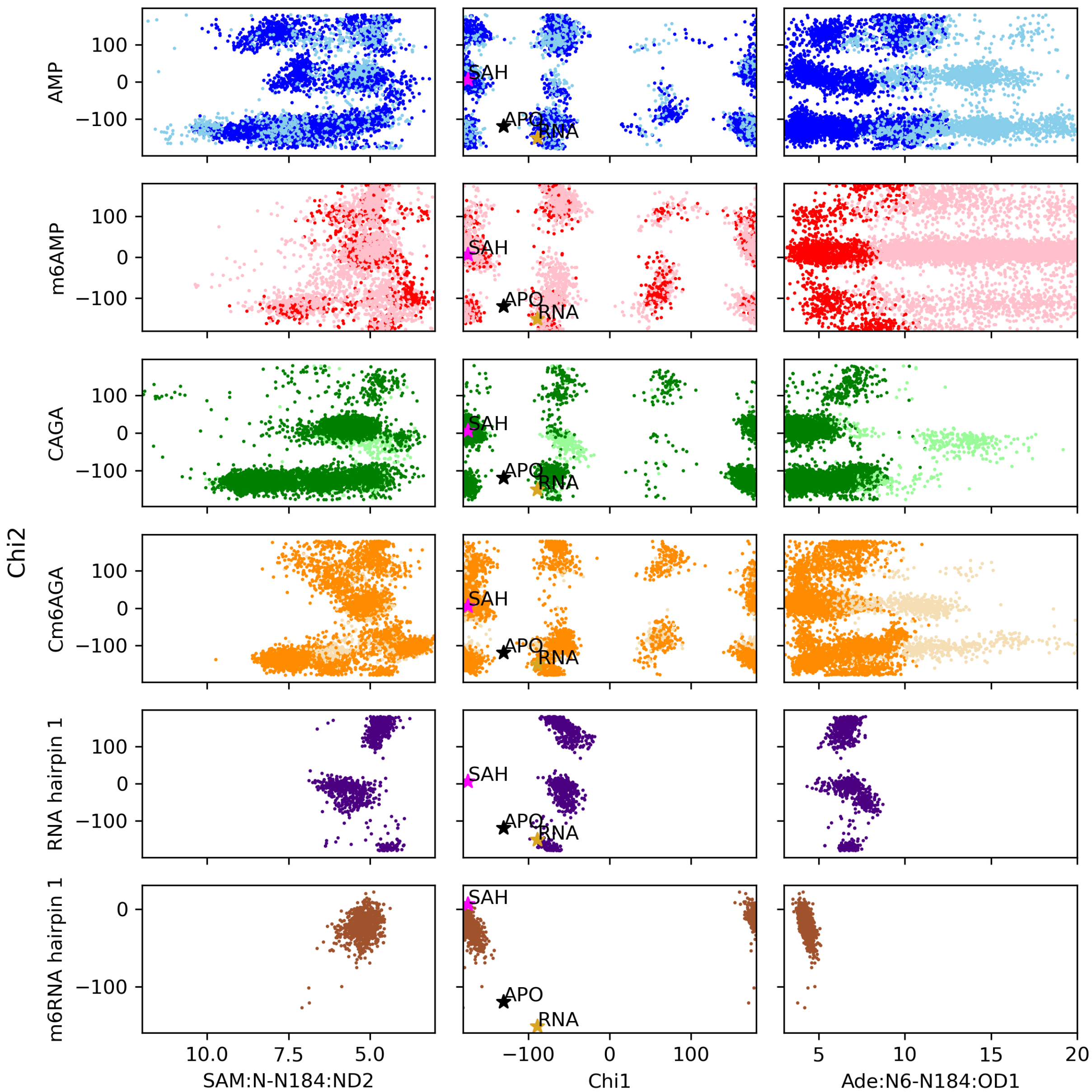

Figure S15: Structural analysis of N184. The central column shows the χ1-χ2 plot for N184. The dihedral angles for the apo (PDB ID: 6B91), SAH-bound (PDB ID: 6GFK), and RNA-bound (PDB ID: 6DU4) are shown as stars. The left column shows the scatterplot of the distance (Å) SAM:N-N184:ND2 vs χ2, while the right shows the distance (Å) Ade:N6-N184:OD1 vs χ2. Darker color indicates bound substrate/product.

2.4 Analysis of simulation convergence

We show here the root mean-squared deviation (RMSD) of the Cα atoms of METTL16 for all systems (Fig. S16). It is noteworthy that there is a small ($\tilde{5}$Å) RMSD for systems where the substrate or product remain bound (shown by darker color in the plot). When there is unbinding, or in the APO system, the RMSD is larger but always below 10 Å. This is expected, when considering the flexibility of METTL16 and especially of its R-loop.

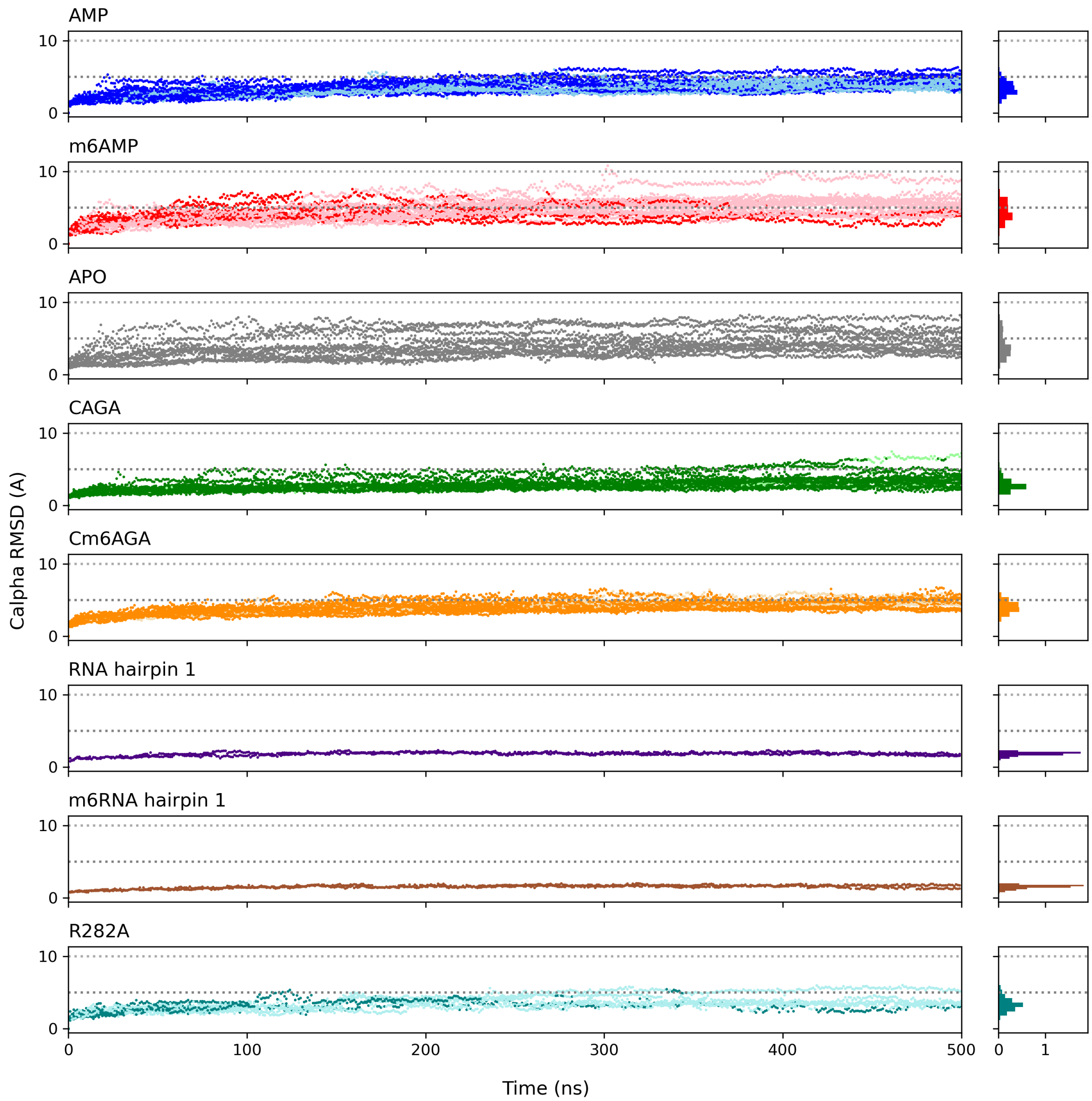

Figure S16: Cα root mean-squared deviation for all systems. Darker color indicates bound substrate or product, i.e., a distance P185:O-A:N6 <7.5 Å.

We also monitor the root mean-squared deviation of the AMP adenine, using as reference the BA2, BA4, and the 6DU4 adenines (Fig. S17). To obtain the reference coordinates, we aligned the crystal structures of the BAs and 6DU4 to the initial frame of each simulation, using the Cα atoms as reference, and then compare the displacement of the AMP or the C**A**GA. This shows that the deviation doesn't vary a lot, independently of the coordinates

used as reference.

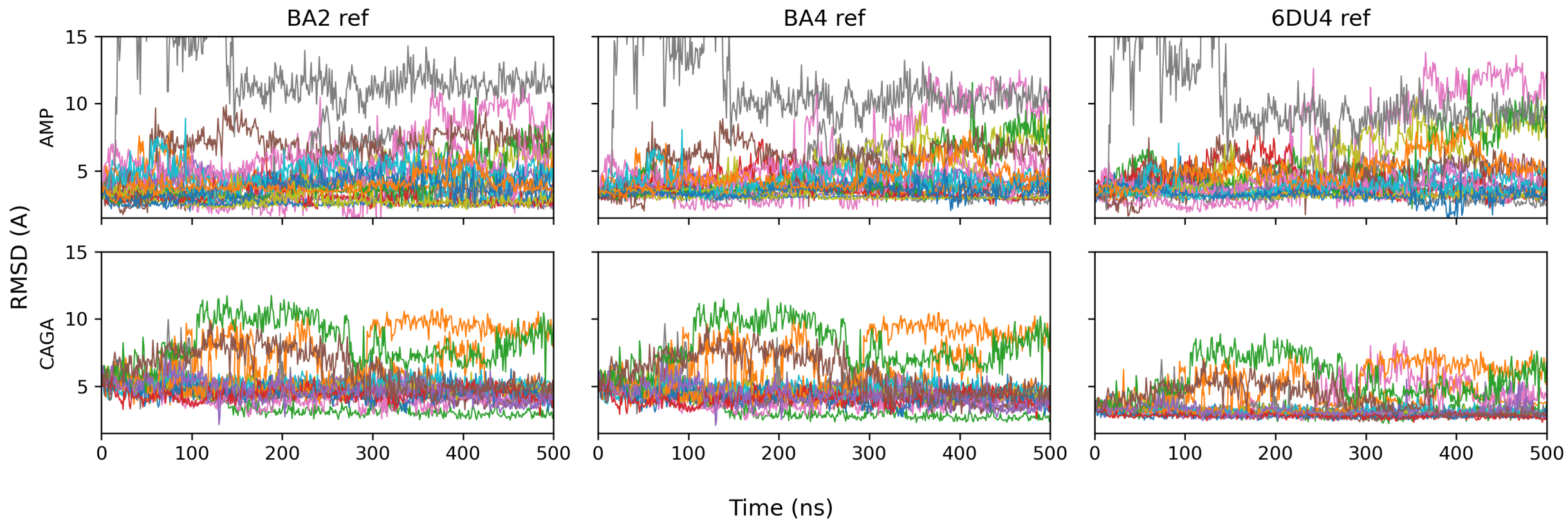

Figure S17: Root mean-squared deviation of adenine atoms against different references coordinates from the crystal structures. Systems were aligned using the Cα atoms of the METTL16, and then the deviations of the relevant adenine were calculated.

3 Quantum Mechanical Calculations

3.1 Gas-phase reaction energetics

We performed QM calculations on truncated models of the S-adenosylmethionine (SAM) cofactor and adenosine (ADE) substrate to evaluate the intrinsic reaction energetics of the enzymatic methyl transfer reaction. The SAM methionine moiety was replaced with an ethyl group, and the adenosine substrate was modeled in both N6-protonated and deprotonated states. Gas-phase calculations were conducted with infinitely separated species using two different density functional theory (DFT) methods, the B3LYP functional[2–4] with the D3 dispersion correction[5] and the ωB97X-D[6] functional, both with the aug-cc-pVDZ basis set.[7,8] To justify the use of semiempirical method for subsequent QM/MM free energy simulations, we also carried out the above calculation using GFN2-xTB method and benchmarked its performance against these DFT methods. All calculations were performed using the ORCA program.[9] We note that GFN2-xTB accurately reproduced the energetic differences between the reaction energetics at two protonation states, validating its capacity to capture the reaction's fundamental energetics (Table S4).

Table S4: Gas-phase reaction energetics of model compounds (in kcal/mol)

System/Method	GFN2-xTB	B3LYP-D3	ωB97X-D
SAM + ADE $\rightarrow$ SAH + m^{6}ADE	0.6	6.8	5.9
SAM + dp-ADE $\rightarrow$ SAH + dp-m^{6}ADE	-140.5	-129.6	-130.5
Difference	-141.1	-136.4	-138.4

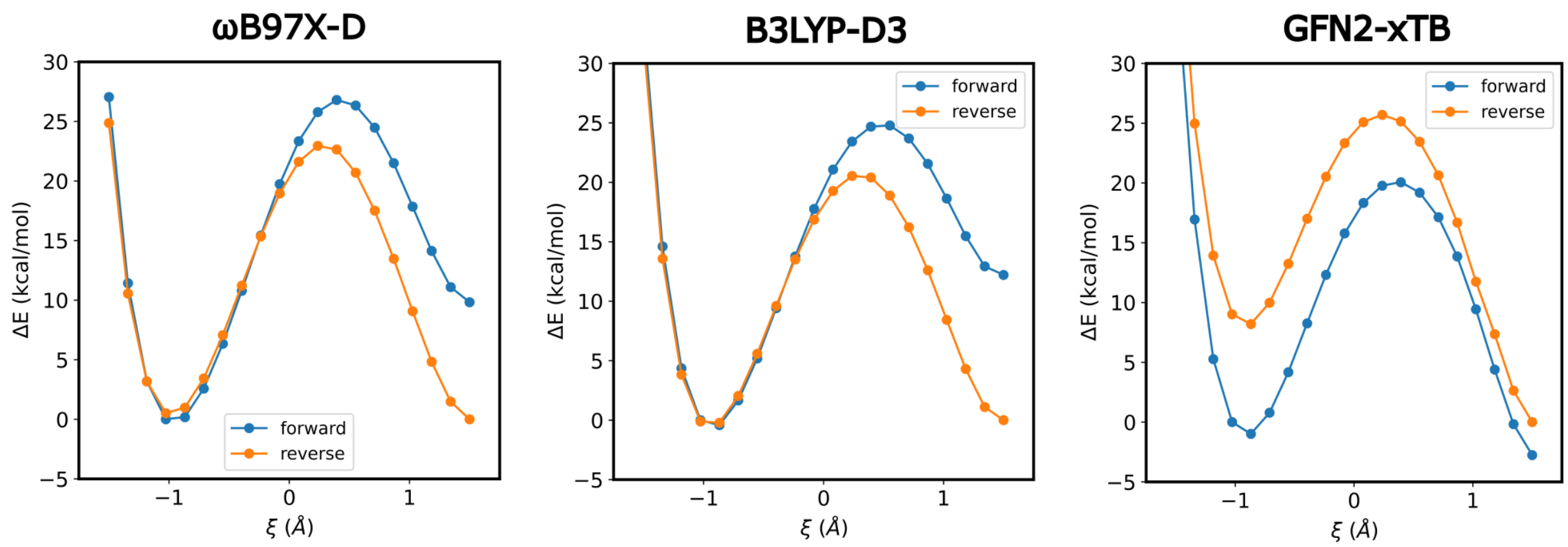

Figure S18: Potential energy surface (PES) scans of the methyl transfer reaction calculated at the DFT and semi-empirical levels of theory. The forward transition is defined as the methyl transfer from SAM to ADE.

Additionally, potential energy surface (PES) scans were performed along the asymmetric stretch of the methyl transfer coordinate using both DFT and semi-empirical methods as described above. To maintain computational efficiency, only the hydrogen atoms and heavy atoms directly involved in the methyl transfer (SD, CE and N6) were relaxed during the scan, while all other heavy atoms remained constrained to their initial equilibrium positions. Despite minor forward/reverse hysteresis resulting from the partially constrained structure, the calculated activation barriers along the reaction coordinate fell consistently within a range of 20–27 kcal/mol across all evaluated methods. These results validate the capacity of GFN2-xTB to accurately capture transition-state energetics alongside the reactant and product states.

3.2 Influence of semiempirical methods and convergence check

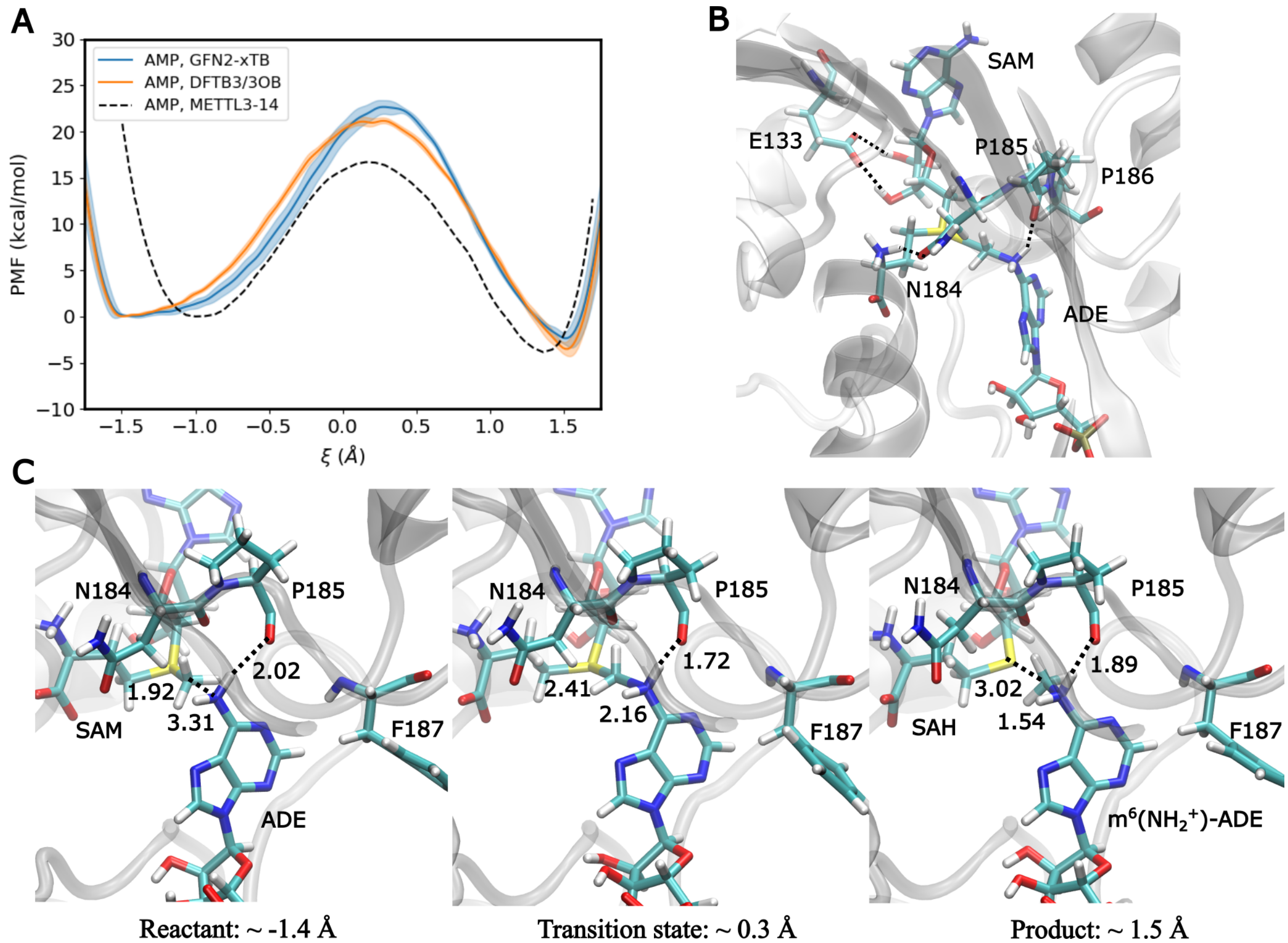

Figure S19: QM/MM free energy simulations results of AMP substrate using DFTB3/3OB as QM method. (A) Estimated PMFs in comparison with GFN2-xTB and prior METTL3-14 results. (B) Components of QM/MM region. (C) Snapshots of active site structure.

To maintain consistency with previous QM/MM studies on the METTL3-14 complex, the METTL16 complex was also simulated using the DFTB3[10] method with the 3OB parameter set[11,12] as the QM level of theory. The QM/MM free energy simulations employed multiple-walker well-tempered metadynamics within the AMBER/PLUMED framework, utilizing an AMP substrate and identical parameters to ensure comparability. The resulting free energy profile (ΔG =-4 kcal/mol; $\Delta G^{\ddagger}$ =21 kcal/mol) and active site configurations were in close agreement with the GFN2-xTB results (ΔG =-3 kcal/mol; $\Delta G^{\ddagger}$ =22 kcal/mol) (Figure S19A). A detailed analysis on the active site structure (Figure S19B) during methyl

transfer reaction confirms that both methods capture similar active site interactions, notably the persistent hydrogen bond between the P185 backbone carbonyl and the adenosine N6 position. Moreover, the poor overlap between the adenine and the F187 side chain implies relatively weak π-π interaction, which agrees with the observation from GFN2-xTB QM/MM simulations. These results validate GFN2-xTB and facilitate a direct comparison between the current findings for METTL16 and prior METTL3-14 data.

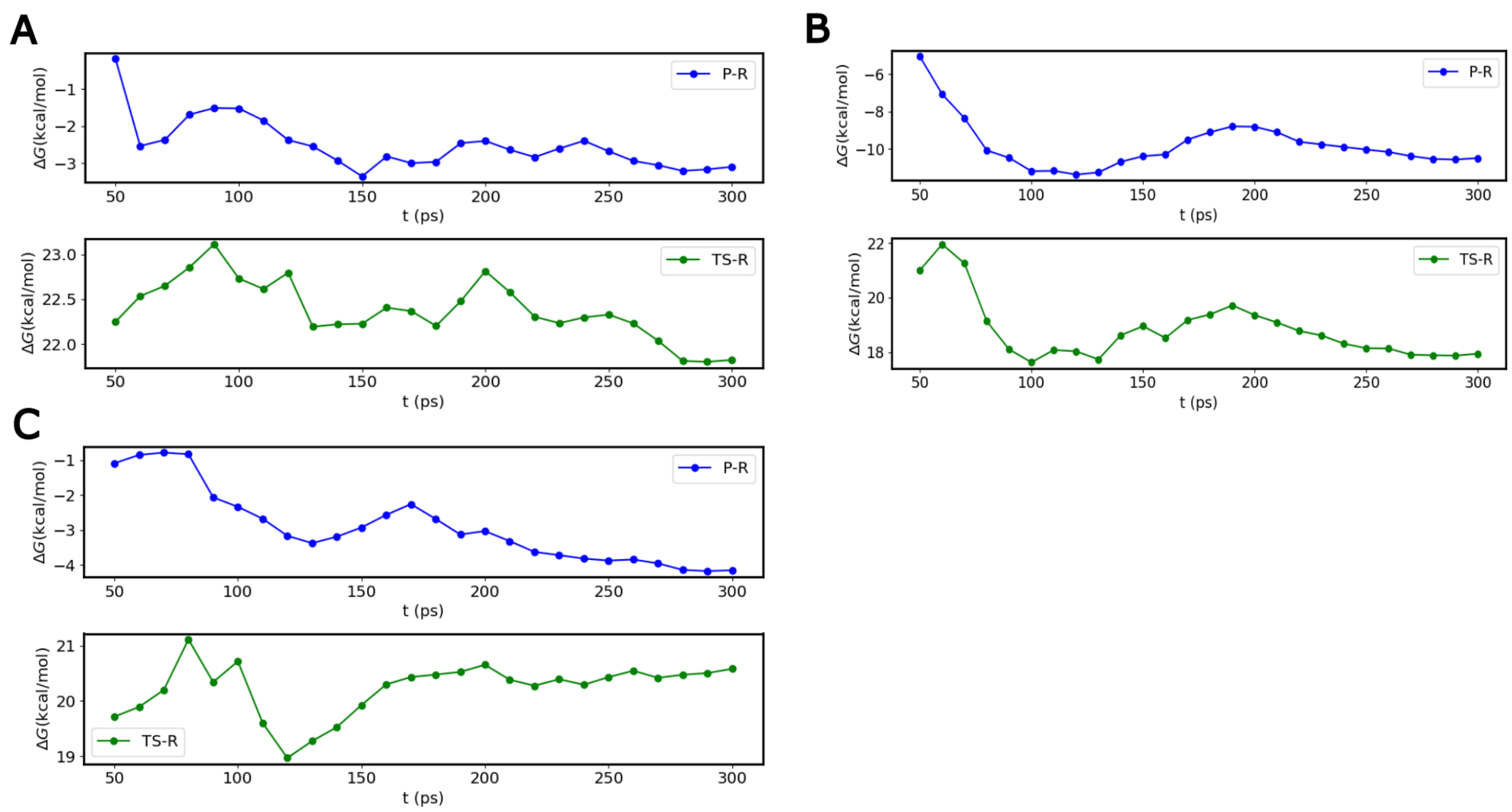

Figure S20: Estimated free energy differences and activation barrier against simulation time per walker in QM/MM simulations. (A) AMP, GFN2-xTB; (B) RNA, GFN2-xTB; (C) AMP, DFTB3/3OB

To assess the convergence of the multiple-walker well-tempered metadynamics simulations, we also monitored the evolution of the potential of mean force (PMF) as a function of simulation time. From the statistics of free energy difference between reactant and product and activation barrier (Figure S20), we concluded that PMF profiles stabilized within 300 ps (per walker, a total of 3 ns), indicating sufficient convergence for the subsequent analysis.

References

(1) Vargas-Rosales, P. A.; Giangreco, G.; Caflisch, A. Disrupting amyloid filaments of tau by means of electric fields. *The Journal of Physical Chemistry B* **2025**, *129*, 9124–9137.

(2) Becke, A. D. Density-functional exchange-energy approximation with correct asymptotic behavior. *Physical Review A* **1988**, *38*, 3098–3100.

(3) Becke, A. D. Density-functional thermochemistry. III. The role of exact exchange. *The Journal of Chemical Physics* **1993**, *98*, 5648–5652.

(4) Lee, C.; Yang, W.; Parr, R. G. Development of the Colle-Salvetti correlation-energy formula into a functional of the electron density. *Physical Review B* **1988**, *37*, 785–789.

(5) Grimme, S.; Ehrlich, S.; Goerigk, L. Effect of the damping function in dispersion corrected density functional theory. *Journal of Computational Chemistry* **2011**, *32*, 1456–1465.

(6) Chai, J.-D.; Head-Gordon, M. Systematic optimization of long-range corrected hybrid density functionals. *The Journal of Chemical Physics* **2008**, *128*.

(7) Kendall, R. A.; Dunning, T. H.; Harrison, R. J. Electron affinities of the first-row atoms revisited. Systematic basis sets and wave functions. *The Journal of Chemical Physics* **1992**, *96*, 6796–6806.

(8) Woon, D. E.; Dunning, T. H. Gaussian basis sets for use in correlated molecular calculations. III. The atoms aluminum through argon. *The Journal of Chemical Physics* **1993**, *98*, 1358–1371.

(9) Neese, F. Software Update: The ORCA Program System—Version 6.0. *WIREs Computational Molecular Science* **2025**, *15*.

(10) Gaus, M.; Cui, Q.; Elstner, M. DFTB3: Extension of the Self-Consistent-Charge Density-Functional Tight-Binding Method (SCC-DFTB). *Journal of Chemical Theory and Computation* **2011**, *7*, 931–948.

(11) Gaus, M.; Goez, A.; Elstner, M. Parametrization and Benchmark of DFTB3 for Organic Molecules. *Journal of Chemical Theory and Computation* **2012**, *9*, 338–354.

(12) Gaus, M.; Lu, X.; Elstner, M.; Cui, Q. Parameterization of DFTB3/3OB for Sulfur and Phosphorus for Chemical and Biological Applications. *Journal of Chemical Theory and Computation* **2014**, *10*, 1518–1537.
